# Antiepileptic drugs’ tolerability and safety – a systematic review and meta-analysis of adverse effects in dogs

**DOI:** 10.1186/s12917-016-0703-y

**Published:** 2016-05-21

**Authors:** Marios Charalambous, Sara K. Shivapour, David C. Brodbelt, Holger A. Volk

**Affiliations:** Faculty of Brain Sciences, UCL Institute of Neurology, University College London, London, WC1E 6BT UK; College of Veterinary Medicine, Iowa State University, Ames, Iowa 50011 USA; Department of Production and Population Health, Royal Veterinary College, Hawkshead Lane, Hatfield, Herts AL9 7TA UK; Department of Clinical Science and Services, Royal Veterinary College, Hawkshead Lane, Hatfield, Herts AL9 7TA UK

**Keywords:** Systematic review, Meta-analysis, Epilepsy, Canine, Antiepileptic drugs, Safety, Side effects

## Abstract

**Background:**

The safety profile of anti-epileptic drugs (AEDs) is an important consideration for the regulatory bodies, owners and prescribing clinicians. Information on their adverse effects still remains limited. A systematic review including a meta-analytic approach was designed to evaluate existing evidence for the safety profile of AEDs in canine patients. Electronic searches of PubMed, CAB Direct and Google scholar were carried out without date or language restrictions. Conference proceedings were also searched. Peer-reviewed full-length studies reporting adverse effects of AEDs in epileptic and healthy non-epileptic dogs were included. Studies were allocated to three groups based on their design. Individual studies were evaluated based on the quality of evidence (study design, study group sizes, subject enrolment quality and overall risk of bias) and the outcome measures reported (proportion of specific adverse effects for each AED, prevalence and 95 % confidence interval of the affected population in each study and comparative odds ratio of adverse effects for AEDs).

**Results:**

Ninety studies, including six conference proceedings, reporting clinical outcomes of AEDs’ adverse effects were identified. Few studies were designed as blinded randomised controlled clinical trials. Many studies included low canine populations with unclear criteria of subject enrolment and short treatment periods. Direct comparisons suggested that imepitoin and levetiracetam might have a better safety profile than phenobarbital, whilst the latter might have a better safety profile than potassium bromide. However, none of these comparisons showed a statistically significant difference. Comparisons between other AEDs were not possible as a considerable amount of studies lacked power calculations or adequate data to allow further statistical analysis. Individual AED assessments indicated that levetiracetam might be one of the safest AEDs, followed by imepitoin and then phenobarbital and potassium bromide; these findings were all supported by a strong level of evidence. The safety profile in other AEDs was variable, but weak evidence was found to permit firm conclusions or to compare their safety to other AEDs.

**Conclusions:**

This systematic review provides objective evaluation of the most commonly used AEDs’ adverse effects. Adverse effects usually appeared mild in all AEDs and subsided once doses and/or serum levels were monitored or after the AED was withdrawn. Although phenobarbital might be less safe than imepitoin and levetiracetam, there was insufficient evidence to classify it as an AED with a high risk of major adverse effects. It is important for clinicians to evaluate both AEDs’ effectiveness and safety on an individual basis before the selection of the appropriate monotherapy or adjunctive AED therapy.

## Background

In human medicine, a plethora of new antiepileptic drugs (AEDs) have been developed over the years for use either as monotherapy or adjunctive therapy [1]. Many of these drugs are now also used in veterinary medicine. This has led to an increase in the arsenal of AEDs used to treat canine epilepsy. As a rule, AEDs are evaluated on the grounds of their effectiveness and safety through clinical trials and experimental laboratory studies before they are approved for use in patients by the regulatory authorities, e.g. the European Medicines Agency (EMA) or the US Food and Drug Administration (FDA) [2]. The safety profile of drugs is an important consideration for their approval by the authorities and use by prescribing clinicians on their clients’ animals [2, 3]. It affects clinicians’ decisions to prescribe specific AED(s), as serious adverse effects can lead to chronic complications or even death. Less serious, but nonetheless important, adverse effects can significantly impact quality of life, leading to systematic illness which may increase the overall cost of treatment [3, 4]. Ultimately, the benefits of an effective AED may be outweighed by its adverse effects, and the latter should be always taken into consideration.

Many potential adverse effects for AEDs have been reported, but the evidence behind the severity of these effects or the likelihood of their occurrence has not been systematically compiled [5, 6]. Randomised clinical trials (RCTs) are a considerable source of evidence for some common or expected adverse effects [4]. However, information about serious, rare, and/or long-term adverse effects can typically be found in studies such as case reports, case series and observational studies [7, 8]. Consequently, the clinician will need to search for information from sources other than RCTs [7, 8]. Identification of all relevant studies can be time-consuming and for a busy practitioner it may be more effective to review this information via a systematic review. Systematic reviews are one of the most powerful and reliable tools to assess the severity and the probability of occurrence of AEDs’ adverse effects across the spectrum of primary literature [9–12].

Although evidence for AEDs’ efficacy has been recently reported and evaluated in a systematic review [13], it has been suggested that, apart from the efficacy, the selection of the appropriate AED should be also largely influenced by its safety profile [14]. To our knowledge there is only one systematic review in the field of canine epilepsy which evaluated the safety profile of a single AED, potassium bromide, across species and aetiology of seizures [15]. However, a systematic review of the adverse effects observed during treatment with any AED(s) in dogs, as well as AEDs’ safety profile comparisons, has not been reported. The aim of this systematic review was to perform an objective analysis of AEDs’ adverse effects in dogs, in order to provide evidence-based information on AEDs’ safety profiles.

## Methods

### Search strategy

The literature search aimed to identify all studies assessing or reporting the adverse effects of an AED in dogs. Specifically, studies were evaluated based on the inclusion criteria below:

- Criterion 1-Type of study: Peer-reviewed studies in English (or translated). Experimental laboratory animal studies, clinical trials, observational and descriptive studies were included.
- Criterion 2-Case definition: For the clinical studies, dogs with IE were included as previously defined [13]. Briefly this required dogs within a certain age range, unremarkable interictal neurological status and diagnostic investigation for seizures. For the experimental laboratory animal studies (ELAS), healthy non-epileptic dogs were also included; for the latter a clear diagnostic investigation or health statement should have been reported in the study to exclude the possibility of underlying diseases.
- Criterion 3-Treatment: Dogs treated with any AED available used in canine IE were included. Doses and serum concentrations of AEDs, frequency of drug administration and treatment period were considered important information to record. Dogs treated with methods other than pharmacological intervention, e.g. homoeopathy methods, surgery, food trials, nerve stimulation, were excluded.
- Criterion 4-Outcome: Studies had to assess or report adverse effects following administration of AED(s) in canine subjects. Studies were conducted either to specifically assess or report AED(s)’ safety (primary evidence studies) or to assess an outcome other than AED(s)’ safety (i.e. efficacy), while also reporting adverse effects (supportive evidence studies). Assessment of the adverse effects should have been performed by the investigators or owner.

Search strategies included use of electronic search engines for publication databases, searching of reference lists of published papers and proceedings of relevant scientific conferences. Electronic databases used were Pub Med (www.ncbi.nlm.nih.gov/PubMed), CAB Abstracts (www.cabdirect.org) and Google Scholar (www.scholar.google.com). Final electronic searches were carried out on 30 February 2015 by the primary and the second author independently, with no date or language restrictions. The search terms used in both search engines were as follows: (dog OR dogs OR canine) AND [(phenobarbital OR phenobarbitone OR primidone OR PBr OR KBr OR potassium bromide OR bromide OR nimodipine OR zonisamide OR ELB138 OR imepitoin OR levetiracetam OR verapamil OR gabapentin OR gaba OR topiramate OR felbamate OR pregabalin) OR [(treatment OR management) AND (epilepsy OR seizures)] OR (anti-convulsant OR anti-seizuring OR anti-epileptic OR AED) AND (safety OR safe OR adverse-effect OR adverse-effect OR effect OR undesirable effect OR tolerability OR toxicity OR drug toxicity OR reactions OR disease). Hand searching for articles from the reference lists of publications and searching major veterinary neurology conference meeting proceedings from 1970 to 2015 and relative textbook chapters was carried out by the primary and second authors independently. Conference proceedings were searched for the Annual Congresses of the European Society and College of Veterinary Neurology (ESVN ⁄ ECVN) and the American College of Veterinary Internal Medicine (ACVIM). Other conference proceedings were searched only if the reference list of identified publications indicated this. All items returned by the search engines, hand searches and correspondence were recorded and entered into the screening process.

### Study selection

Restrictions based on publication date or language were not imposed. Studies written in non-English language were assessed initially based on an English translation (Google Translate software) and then verified by a veterinarian fluent in the language of publication.

A two-stage screening process was used [13] and the process was performed by the primary author. Firstly, studies of relevance to the systematic review objectives were identified (stage 1) and, secondly, studies likely to provide evidence of the highest available quality and sufficient detail for assessing the outcome measures and methodology were selected (stage 2). Stage 1 of the screening process identified from the total search results any studies that: (a) fulfilled inclusion criterion 1 and (b) reported findings related to the adverse effects and safety of AEDs administered in dogs. Stage 1 assessment evaluated the retrieved papers’ titles and abstracts only. At stage 2, papers were selected for full data extraction according to the inclusion criteria 2, 3 and 4 and were evaluated in detail on the grounds of the quality of evidence and outcomes by MC.

### Assessment of quality of evidence

Blinded RCTs (bRCTs) and blinded randomised ELAS (bRELAS) were considered most likely to produce higher quality evidence, followed by non-blinded RCTs (nbRCTs) and non-blinded randomised ELAS (nbRELAS), then non-randomised clinical trials (NRCTs) and non-randomised ELAS (NRELAS), uncontrolled clinical trials (UCTs) and uncontrolled ELAS (UELAS), cohort, case–control and cross sectional studies and lastly case series and reports [16–18]. Accordingly, the studies were allocated based on their design to one of three groups, i.e. bRCTs, bRELAS, nbRCTs and nbRELAS (first group), NRCTs, NRELAS, UCTs, UELAS, cohort, case–control and cross-sectional studies (second group) and case series and reports (third group).

As a general rule, the studies in the first group (bRCTs and bELAS in particular) were considered to provide higher quality evidence, followed by the studies in the second and third group. In addition, a three-part system of evidence quality assessment to indicate the strengths and weaknesses of each study within each group was used [13, 19]: (a) study group sizes, (b) subject enrolment quality and (c) overall risk of bias based on Cochrane [20] and Syrcle’s [21] ‘risk of bias’ assessment tool in order to provide an indicator of confidence associated with the findings of each study. For instance, bRCTs or bRELAS with large group sizes, clear inclusion criteria, thorough diagnostic investigations and low overall risk of bias were considered to provide the highest available quality of evidence.

### Study group sizes

This characteristic was categorized for each study using the following system [13, 19]: (a) >50 subjects per group (‘good’ number of subjects), (b) 20–50 subjects per group (‘moderate’ number), (c) 10–19 subjects per group (‘small’ number) and (d) <10 subjects per group (‘very small’ number).

### Assessment of subject enrolment quality

Data on investigations to reach the diagnosis of IE were retrieved to evaluate the quality of subject enrolment in each study as ‘well characterized’, ‘fairly characterized’, ‘poorly characterized’ or ‘unclear.’ Well characterized diagnoses were defined as diagnostic investigations that included clinical signs and thorough test results consistent with the diagnosis of IE; specifically, the signalment, the absence of neurological deficits between the ictal phases, unremarkable routine biochemical and haematological blood tests and imaging results (including brain MRI and/or CT) and/or normal cerebrospinal fluid (CSF) analysis for all cases of the study. Fairly characterized, used for intermediate situations, were defined as diagnostic investigations that were based on signalment, clinical examination and basic diagnostic investigation (i.e. blood tests only), with only some study cases having had advanced brain imaging and/or CSF analysis. Poorly characterized were defined as diagnostic investigations that were based on signalment, clinical examination and/or basic diagnostic investigation (i.e. blood tests) only. Unclear related to reports where the approach to diagnosis of IE was not clearly stated (e.g. when clinical signs were not stated and insufficient or no details of diagnostic tests were provided or when dogs with IE were included without reporting details on diagnostic investigation).

For the ELAS, which included non-epileptic healthy animals, ‘clearly characterized’ were the studies that defined diagnostic investigations and thorough test results to exclude any systemic illness; ‘unclear’ were characterized when diagnostic investigations to rule out diseases were not clearly stated or when dogs were included and considered healthy without reporting details on diagnostic procedures.

### Assessment of overall risk of bias

The overall risk of bias in the clinical trials was assessed based on the criteria of the Cochrane ‘risk of bias’ assessment tool [20]. Syrcle’s ‘risk of bias’ assessment tool [21] was used to assess the overall risk of bias in ELAS. The latter tool is an adapted version of the Cochrane one and was designed to facilitate critical appraisal of evidence from ELAS.

Each of the following study components was categorized as presenting a ‘high’, ‘low’ or ‘unclear’ risk of introducing bias to the study findings: random sequence generation, allocation concealment, blinding of participants and personnel, blinding of outcome assessment, completeness of outcome data, selective reporting of outcomes and other sources of bias. For ELAS, two further components-random housing and baseline characteristics of dogs - were also assessed and mentioned as part of the “other sources of bias” section. Case series and reports as well as observational studies were considered to be of high overall risk of bias.

### Level of the studies’ evidence

The level of evidence provided for the safety profile of each AED was based on the overall quality of evidence of the studies. The level of evidence was allocated according to a previous similar system [13, 19] which was extensively modified for the needs of the current study: ‘strong’ evidence was provided for the safety profile when at least one bRCT and/or bRELAS reported or assessed the adverse effects of an AED; ‘weak’ evidence was provided for the safety profile when bRCTs and/or bRELAS were not available.

### Assessment of outcome measures

The outcome measure of this study was the evaluation of the safety profile of AED(s) administered in dogs. The adverse effects were organized by body system (e.g. neurological, gastro-intestinal, dermatological, etc.) and types, including type I (dose dependent and predictable) and type II (idiosyncratic-dose independent and unpredictable). Different terms used by the studies but describing the same adverse effects (e.g. drowsiness and somnolence, wobbly gait and ataxia, lethargy and sedation, etc.) were considered synonymous and only one term was selected for use in the analysis. The outcome measure was assessed according to the methods below:

### Proportion of specific adverse effects for each AED

This was expressed as a percentage and calculated for each AED by dividing the number of studies that reported a specific adverse effect by the total number of the studies for this AED. If an AED was used as a monotherapy and adjunctive therapy, further calculations were also performed for each sub-category.

### Prevalence and 95 % confidence interval of the affected population in each study

Prevalence was expressed as a percentage and calculated for each study by dividing the number of subjects that developed adverse effects during the specified study period by the total size of the study population. The 95 % confidence interval (95 % CI) of the proportion of study animals that developed adverse effects related to the AED(s) was calculated by standard methods [22]. This was used as a further indicator of an AED’s safety profile. If the 95 % CI of affected dogs (based on 95 % CI calculations) were ≥ 50 %, then it was considered that the majority of the study population experienced adverse effects.

For each study, the period of treatment, AED’s doses and serum levels were reported with the aim to evaluate the association of these values with the prevalence of each AED’s adverse effects.

### Statistical analysis

For the comparison groups’ studies, a further approach was conducted to identify statistical differences between studies with regards to reported adverse effects. For each AED study, the total number of patients experiencing adverse effects and/or the number of patients experiencing specific adverse effects (e.g. sedation, ataxia, polyuria, etc.) in all therapeutic groups were retrieved. The odds ratio (OR) was then estimated in order to indicate the increased or decreased odds of observing a specific adverse effect(s) in total for an AED compared to its control group (comparison AED or placebo or untreated animals). Statistical analysis was undertaken following the guidelines of the Handbook of the Cochrane Collaboration 5.0. The OR for dichotomous data was calculated using the random-effects model in Review Manager 5.3. Heterogeneity between studies was calculated using the Chi square test and was considered to be heterogeneous when P ≤ 0.1. I^2^ values of no more than 25, 26 to 74 % and no less than 75 % were considered as “low”, “moderate” and “high” heterogeneity, respectively. Associations were considered to be statistically significant at P < 0.05. P values between 0.05 and 0.1 were considered as statistical trends of potential interest.

## Results

### Description of studies

By 29 December 2015, the search strategy had identified a total of 368 unique citations; 347 from the electronic searches of PubMed, CAB Abstracts, Google Scholar and manual searches from the publications’ reference lists, 16 from manual searching of major conference proceedings and 6 unpublished studies included as part of published data. Two hundred ninety two items fulfilled stage 1 screening criteria. Of these, 90 final studies (published between 1981 and 2015) also fulfilled stage 2 selection criteria and were thus selected for review.

The vast majority of studies were allocated in the second (i.e. non-blinded, non-randomised and uncontrolled studies) and third (i.e. retrospective case series and reports) group. A few studies included more than one sub-study (i.e. a clinical trial and/or ELAS and/or retrospective case series part); accordingly, such studies were included in more than one group. Therefore, study designs represented were five bRCT [23–27], two nbRCT [28, 29] and seven nbRELAS [25, 30–35] in the first group, six NRCTs [36–41], 11 NRELAS [42–52], 22 UCTs [44, 48, 53–71], six UELAS [34, 72–76] and one cross sectional study [3] in second group, and 19 retrospective case series [77–95] and 16 case reports [96–111] in the third group. In addition, five unpublished studies described adverse effects and were reported briefly in EMA report; thus all these were considered as one study [112] and were not included in any category as there was insufficient information as far as their design was concerned.

Overall, the 90 selected studies reported 12 AEDs. In all studies but one [43], the AEDs were orally administered. Within each study, one or more AEDs were evaluated as a monotherapy and/or adjunct to other AEDs.

### Disease characterisation

In the majority of the studies, the inclusion criteria for diagnosing IE were not well characterized. According to the described grading system for subject enrolment quality, 16 studies [27, 33, 53, 61, 63, 64, 68, 80, 81, 90, 96, 97, 100, 101, 110, 111] enroled treatment groups of well characterized IE, 13 studies [3, 44, 48, 54–57, 62, 66, 67, 77–79] enroled treatment groups of fairly characterized IE, and 14 studies [23–26, 39, 58, 59, 65, 74, 82, 84, 88, 108, 109] enroled treatment groups of poorly characterized IE. In 26 studies [28, 29, 36–38, 50, 60, 69, 70, 75, 83, 85–87, 89, 91–94, 98, 99, 102–106], the diagnostic procedures for enrolment of cases with IE were unclear.

As far as the ELAS including healthy animals were concerned, eight [31, 36, 45, 46, 50–52, 73] enroled treatment groups of clear and 14 [25, 30, 32, 34, 35, 37, 38, 42, 43, 47, 49, 72, 76] enroled treatment groups of unclear or unknown diagnostic investigation for ruling out other diseases. In one report, a dog was non-epileptic and was treated with phenobarbital and chlomipramine due to anxiety and aggression, but the diagnostic investigation for this was unclear [107].

### Study group sizes

The vast majority of studies reported the total number of dogs evaluated. The majority of studies evaluated small or very small study size groups. Thirteen studies [25, 26, 40, 50, 62, 69–71, 75, 82, 88, 90, 113] evaluated groups with a good number of dogs, 13 studies [23, 24, 32, 37, 39, 65, 74, 77, 79, 80, 91, 94, 95] evaluated groups with a moderate number of dogs, 26 studies [3, 28, 34, 36, 38, 44–46, 48, 53, 54, 56–61, 63, 64, 66, 70, 75, 81, 83, 84, 114] evaluated groups with a small number of dogs and 38 studies [33–35, 39, 42, 43, 51–53, 55, 67, 68, 72, 73, 76, 78, 85, 87, 89, 92, 93, 96–100, 102–111, 115, 116] evaluated groups with a very small number of dogs. In two studies, the study group size was unclear [47, 49].

### Signalment and baseline characteristics of study subjects

Baseline characteristics (such as breed, age and sex) of total enroled dogs were reported to some extent for all 90 studies. Clear presentation of statistical comparison of intervention groups with respect to signalment and baseline disease characteristics was not commonly encountered.

In all studies reporting baseline data, the recruited dogs represented multiple breeds, both sexes and a wide range of ages at study entry (median 5, mean 4, range 0.5-7 years). Major affected breeds were crossed-breeds and pure breeds such as Labrador and Golden Retrievers followed by German Shepherd dogs, Beagles, Boxers and Poodles. In the majority of the studies more males were affected compared to females, though these differences were not evaluated statistically.

### Methodological quality of included studies

**Fig. 1:**
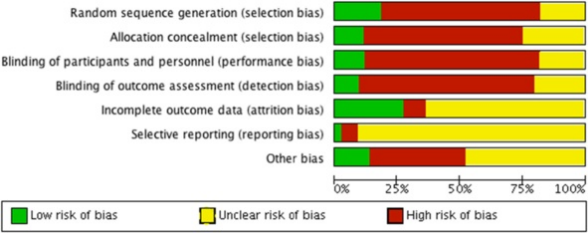
Risk of bias. Risk of bias assessment presented as percentages across all included studies

The vast majority of studies revealed high and/or unclear risk of bias for all the components (Fig. 1). As stated in the methods, retrospective case series and reports were not included in the methodological quality assessment as these were considered to be at an overall high risk of bias.

#### Randomization and allocation concealment

Studies in group A used randomization to allocate the dogs and were considered to provide a low risk of bias. Eight studies [25, 28, 29, 32–35, 68] did not offer enough detail to confirm that allocation concealment was performed. Five studies stated that randomization was concealed without further details. Two studies [23, 27] assigned by random blocking (random allocation to blocks of 10 and 6 respectively). One study [27] used pre-defined randomization lists under double-blinded conditions. One study [31] used drawing lots and two studies [24, 30] used a computer-generated list of random numbers.

#### Blinding of outcome assessment

Only in five studies [23–27], in group A, blinding was clearly described; these were also considered to be at low risk. In these five studies, blinding was applied to all participants, personnel and outcome assessment. In one of them [23] all but the primary investigator were blinded.

#### Incomplete outcome data

Ten studies presented outcome data from all enroled dogs in the treatment group to which they were originally allocated and there were no losses between enrolment and evaluation [30, 33, 42, 51, 53, 59, 61, 67, 73, 78]. The same studies were considered to be at low risk of bias. In two studies, it was unclear whether all dogs completed the study, as inadequate information was provided [60, 64]. Across the remaining studies, there were dogs that were euthanized or excluded due to poor seizure control, owner request or for unidentified reasons; thus there were losses between the initial study populations and the final number of the dogs.

#### Selective reporting

It was difficult to assess selective reporting as study protocols were not sought beyond the information published. In two reports [29, 112] further information was attempted to be retrieved but complete protocols were never obtained.

#### Acknowledgment of other sources of bias

Twelve studies reported financial support [24, 26–28, 41, 44, 53, 58, 61, 68, 73, 79] but there was not adequate evidence to support whether this biased the results. One study [54] clearly mentioned that there was no financial support, while the remaining studies failed to report financial support.

In two studies [28, 78] the statistical analysis was not clarified. In one study [25], many dogs were excluded from both groups mainly for treatment-related reasons (post-randomisation bias). Six studies [29, 49, 60, 64, 70, 86] were conference abstracts, thus no further information could be retrieved. One dog in one study [66] and two dogs in two studies [28, 63] were diagnosed with symptomatic epilepsy (i.e. a cause was identified); this could potentially affect the final results on AED safety profile. Conflict of interest was clearly stated in one study [25].

In the ELAS, specifically, nine studies [25, 30, 33, 34, 42, 44, 51, 52, 68] reported details for the experimental dogs’ housing. Random housing of the dogs occurred in all but nine studies [34, 35, 37, 42–44, 49, 72, 73]. The baseline characteristics of the dogs were reported in nine studies [25, 30, 33, 42, 44, 51, 52, 68, 73] and were similar for all the experimental groups in seven studies [25, 30, 33, 42, 44, 51, 52].

### AEDs safety profile

#### A) Safety profile for each AED individually

**Fig. 2:**
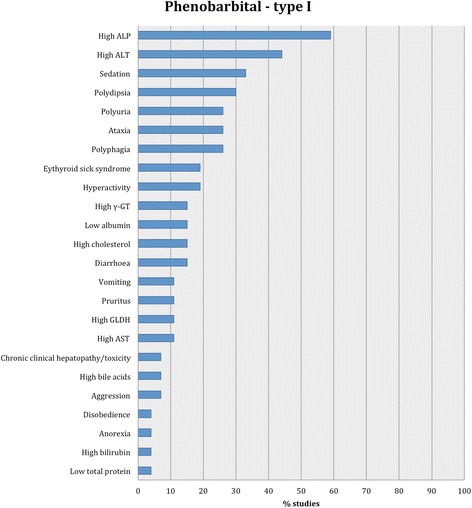
Proportion of specific type I adverse effects for phenobarbital. Each adverse effect represents the percentage of studies that reported this specific adverse effect for phenobarbital monotherapy

**Fig. 3:**
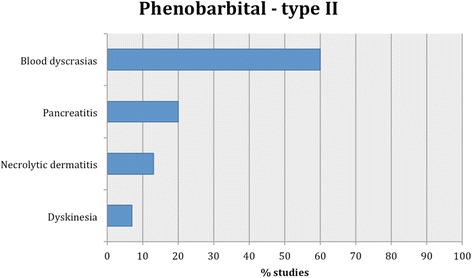
Proportion of specific type II adverse effects for phenobarbital. Each adverse effect represents the percentage of studies that reported this specific adverse effect for phenobarbital monotherapy

**Fig. 4:**
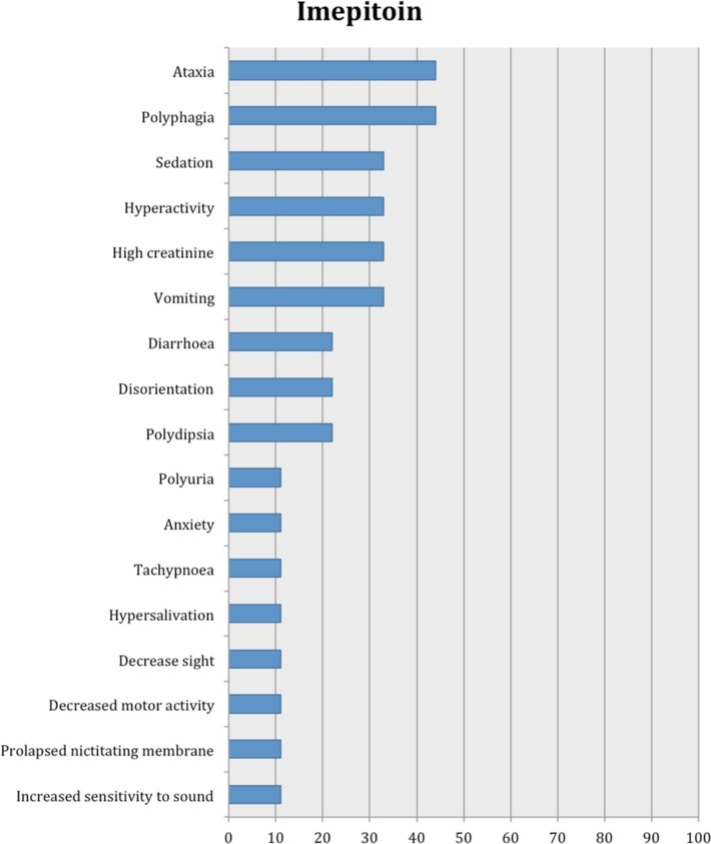
Proportion of type I adverse effects for imepitoin. Each adverse effect represents the percentage of studies that reported this specific adverse effect for imepitoin monotherapy

**Fig. 5:**
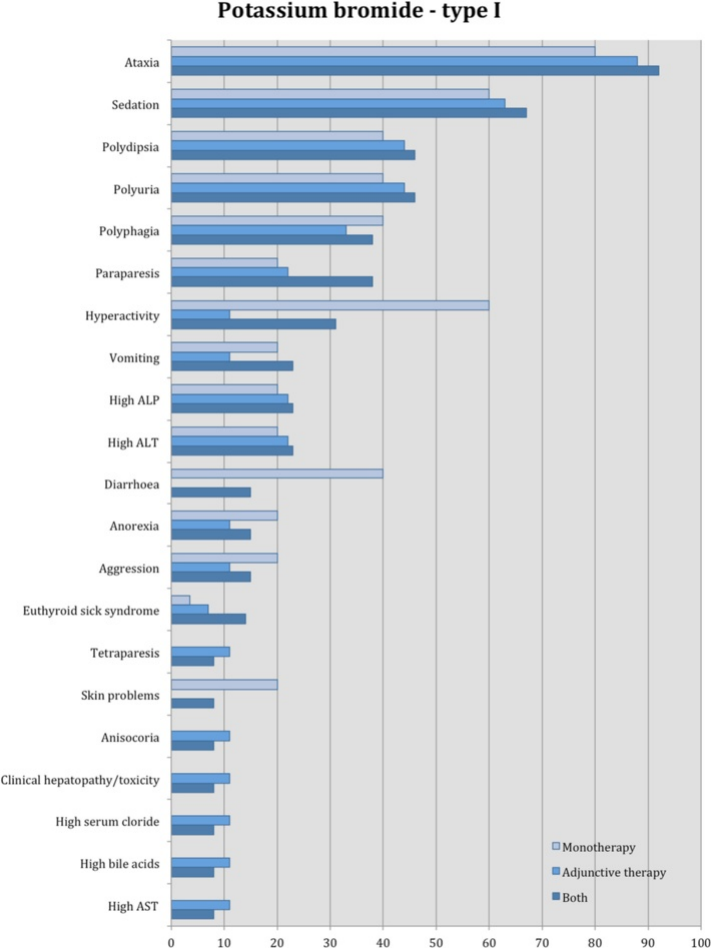
Proportion of specific type I adverse effects for potassium bromide. Each adverse effect represents the percentage of studies that reported this specific adverse effect for potassium bromide monotherapy and adjunctive therapy

**Fig. 6:**
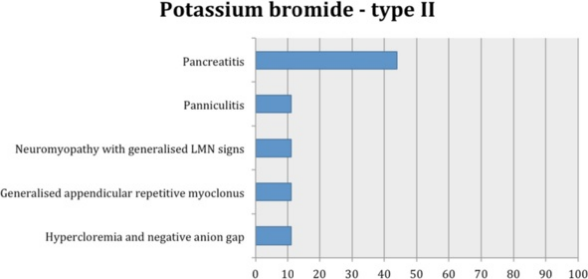
Proportion of specific type II adverse effects for potassium bromide. Each adverse effect represents the percentage of studies that reported this specific adverse effect for potassium bromide monotherapy and/or adjunctive therapy

**Fig. 7:**
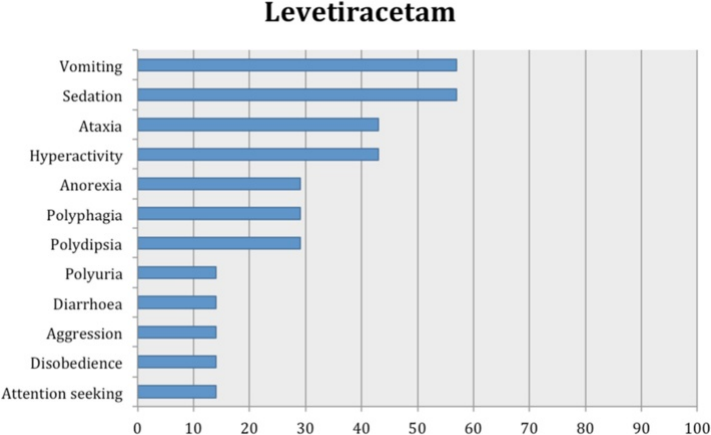
Proportion of specific type I adverse effects for levetiracetam. Each adverse effect represents the percentage of studies that reported this specific adverse effect for levetiracetam monotherapy and/or adjunctive therapy

**Fig. 8:**
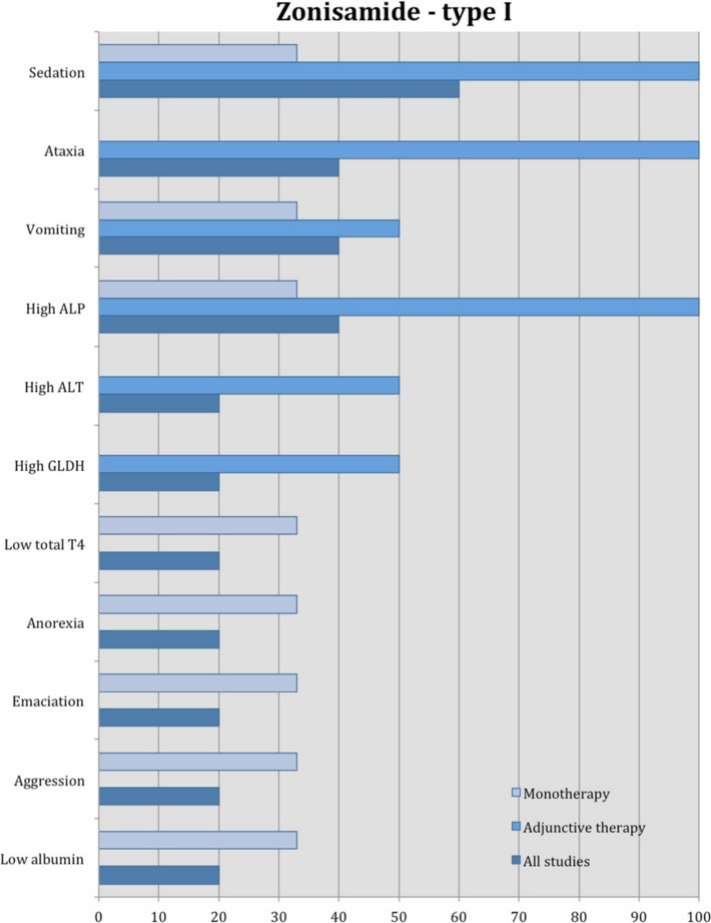
Proportion of specific type I adverse effects for zonisamide. Each adverse effect represents the percentage of studies that reported this specific adverse effect for zonisamide monotherapy and adjunctive therapy

**Fig. 9:**
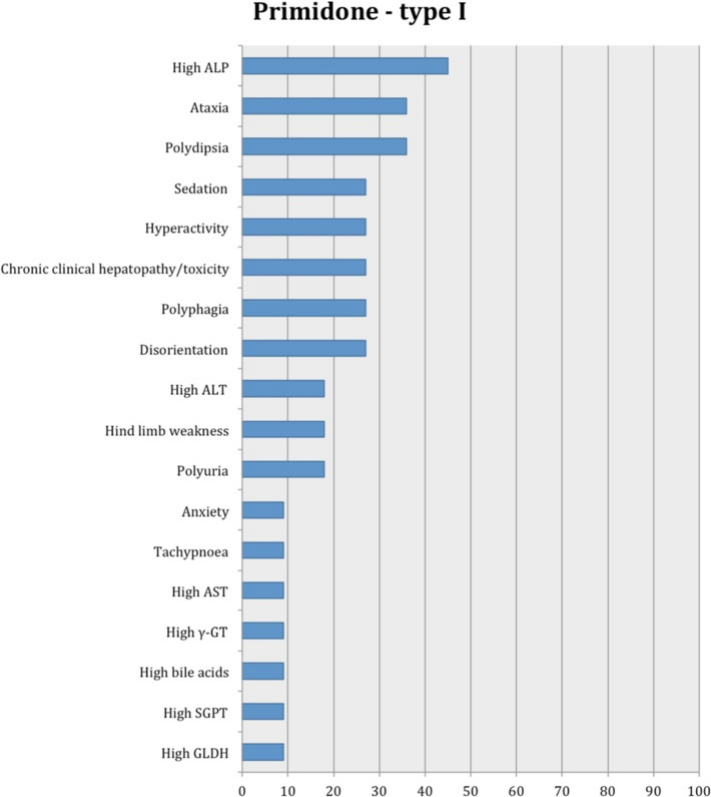
Proportion of specific type I adverse effects for primidone. Each adverse effect represents the percentage of studies that reported this specific adverse effect for primidone monotherapy

**Fig. 10:**
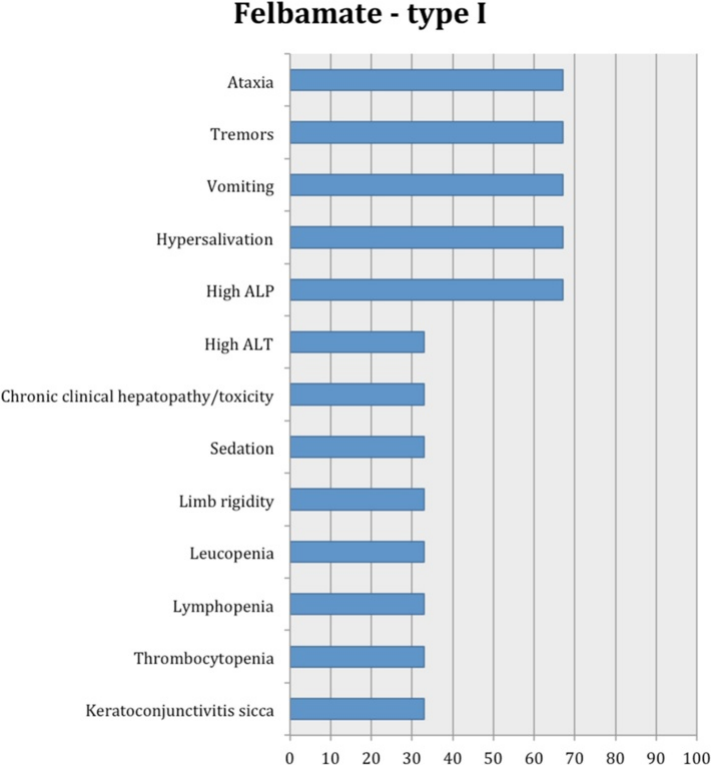
Proportion of specific type I adverse effects for felbamate. Each adverse effect represents the percentage of studies that reported this specific adverse effect for felbamate monotherapy and/or adjunctive therapy

**Fig. 11:**
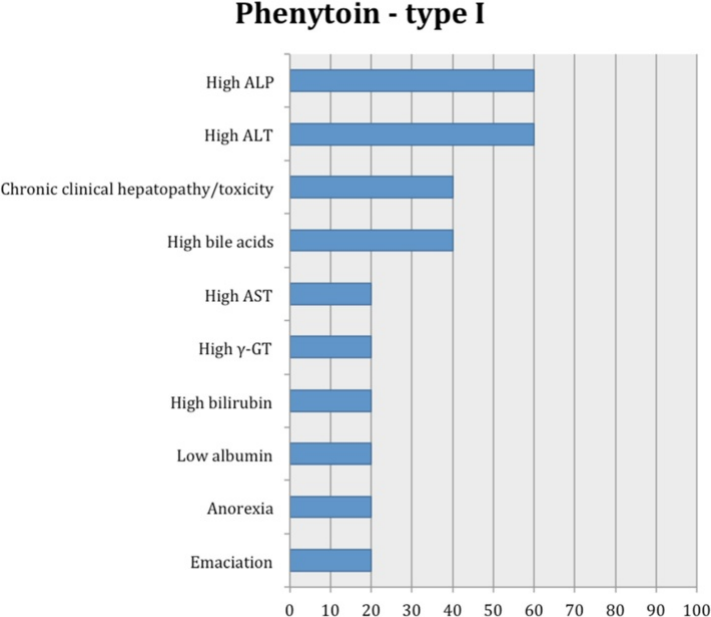
Proportion of specific type I adverse effects for phenytoin. Each adverse effect represents the percentage of studies that reported this specific adverse effect for phenytoin monotherapy and/or adjunctive therapy

Proportions of adverse effects for each AED are summarized in the text and presented in Figs. 2, 3, 4, 5, 6, 7, 8, 9, 10, 11. Details of doses and serum concentrations of AED(s), period of treatment as well as prevalence of adverse effects and 95 % CI of the proportion of affected cases (included type and most frequently occurred) for each study are summarized in the text and provided in detail in Tables 1, 2, 3, 4, 5, 6, 7.

**Table 1:** Details of number of dogs, 95 % CI affected cases, AED doses and serum levels, treatment period and adverse effects

Studies	AED	No of dogs treated	Prevalence	95 % CI affected cases	Doses of AEDs	Serum levels of AEDs	Treatment period	Body system affected and adverse effects	Most common adverse effects	Adverse effect type
Boothe et al. 2012	PHB	20	78.5 %	60.5 %–96.5 %	mean, 4.11+/−1.1; range, 3.9–4.9 mg/kg PO BID	mean, 27+/−6; range, 12.4–36 μg/mL	6 m	Neurological (ataxia, hyperactivity, sedation), GI (vomiting, diarrhea, PP), PU, PD, ClinPath (increased ALP, decreased albumin)	ataxia, sedation, increased serum ALP, decreased albumin	I
Heynold et al. 1997	PHB	37	35 %	19.6 %–50.4 %	mean, 2.5 mg/kg PO BID	range, 15–40 μg/ml	mean, 50; range, 8–108 m	Neurological (ataxia, sedation, aggression), GI (PP), Dermatological (itching)	sedation	I
Löscher et al. 2013	PHB	8	NA	NA	range, 10–40 mg/kg PO SID	14.7 μg/ml	2.5 m	Neurological (sedation, ataxia), PU, PD	sedation, ataxia, PU, PD	I
Gaskill et al. 2000	PHB	22	32 %	12.5 %–51.5 %	At 3 w: mean, 3.6+/−1.3; range, 1.3–6.0 mg/kg At 6 m: mean, 3.7+/−1.4; range, 1.3–8.3 mg/kg At 12 m: mean, 3.7+/−1.6; range, 1.3–8.3 mg/kg PO SID	At 3 w: mean, 58.6+/−15.0; range, 33–85 mmol/L At 6 m: 62.5+/−25.7; range, 8–120 mmol/L. At 12 m: mean, 62.2+/−23.51; range, 11–116 mmol/L	12 m	Endocrine (decreased total T4 levels, increased TSH levels, normal TSH stimulation test)	euthyroid sick syndrome	I
Steinberg 2004	PHB (monotherapy prior to the addition of other AEDs)	14	26.6 %	4.8 %–52.1 %	NA	PHB: mean, 32.1+/−14.4 μg/ml.	median, 17; range, 3.3–58.5 m	GI (chronic hepatotoxicity)	chronic hepatotoxicity	I
von Klopmann et al. 2006	PHB	34	68 %	52.3 %–83.7 %	NA	NA	NA	Endocrine (decreased total T4 levels, normal TSH levels, normal TSH stimulation test)	euthyroid sick syndrome	I
Chang et al. 2006	PHB	11	92.5 %	76.9 %–108.1 %	NA	NA	median, 18; range, 3–72 m	Neurological (ataxia, hyperactivity, sedation), GI (vomiting, diarrhea, PP), Dermatological (itching), PU, PD	PD, PU, PP, sedation, hyperactivity	I
Tipold et al. 2014	PHB	110	57.3 %	48.1 %–66.5 %	range, 2–6 mg/kg PO BID	<45 μg/mL	5 m	Neurological (sedation), GI (PP, diarrhea), PU, PD, ClinPath (increased ALP, γ-GT, ALT and GLDH)	sedation, PP, PU, PD	I
Fredso et al. 2015	PHB	6	93 %	70 %–114 %	median, 2.7; mean, 3; range 2.2–3 mg/kg PO BID	median, 77; mean, 77.3; range 55–111 μmol/L	2–12 m	Neurological (sedation, ataxia, hyperactivity, disobedience), GI (PP), PU, PD	PD, PP	I
Schwartz-Porsche et al. 1985	PHB	15	93 %	80.1 %–105.9 %	range, 5–17 mg/kg PO SID	range, 19–57 μg/ml	mean, 15; range, 7.3–32 m	Neurological (sedation, ataxia), GI (PP), PD, ClinPath (ALT, ALP, GLDH)	ataxia, sedation, PP, PD	I
Gaskill et al. 2005	PHB	12	NA	NA	median, 5; range, 2.1–12.9 mg/kg PO SID	mean, 22.8; range, 9.7–44.2 μg/ml	median, 20.4; range, 4–78 m	ClinPath (increased ALT, ALP)	increased ALT, ALP	I
Farnbach et al. 1984	PHB	42	2.4 %	−2.2 %−7.2 %	range, 0.3–19.9 mg/kg PO SID	mean, 24.3; range, 6.5–81.3 μg/ml	NA	Neurological (hyperactivity)	hyperactivity	I
Gaskill and Kimber 2010	PHB	30	80 %	65.7 %–94.3 %	NA	NA	12 m	Neurological (ataxia, sedation, hyperactivity, aggression), GI (PP, anorexia, vomiting, diarrhoea), Dermatological (skin problems), ClinPath (increased ALP, ALT, lipase), PU, PD	PP, PU, PD, vomiting, skin problems, hyperactivity	I
Aitken et al. 2003	PHB	95	40 %	30.1 %–49.8 %	<2–> 10 mg/kg PO SID	<65–> 120 μmol/l	<3–> 12 m	ClinPath (increased ALT, ALP, γ-GT, GLDH, cholesterol, bile acids)	increased ALP, ALT, GLDH	I
Dayrell-Hart et al. 1991	PHB	18	NA	NA	median, 10.4; range, 3.1–27 mg/kg PO SID	mean, 49.7; range, 16–60 μg/ml (12 dogs had >40)	median, 39; range, 5–82 m	GI (hepatotoxicity) (also all dogs were ataxic and sedated)	NA	I
Andrik et al. 2010	PHB	30 (15 epileptic and 15 non-epileptic)	NA	NA	Epileptic dogs: 2 mg/kg PO BID (increased if necessary) Non-epileptic dogs: Initially at 2 mg/kg PO SID, then increased at 8 mg/kg PO SID	NA	Epileptic dogs: range, 12–60 m Non-epileptic dogs: 5 m	GI (chronic hepatotoxicity), ClinPath (increased ALP, ALT, AST, total bilirubin, decreased albumin and total protein)	increased ALT, ALP	I
Litchfield et al. 1972	PHB	4	NA	NA	range, 5–40 mg/kg IV SID	NA	0.5 m	ClinPath (increased ALP)	NA	I
Foster et al. 2000	PHB	Experimental dogs: 6 Epileptic dogs: 10	70 %	Experimental dogs: 0 % Epileptic dogs: 41.6 %–98.4 %	Experimental dogs: mean, 6 mg/kg; range, 5.9–6.4 mg/kg PO SID Epileptic dogs: range, 3.9–14.4 mg/kg PO SID	Experimental dogs: mean, 63+/−15, range, <65–194 μmol/L Epileptic dogs: mean, 110; range, 72–171 μmol/L	Experimental dogs: 3 m Epileptic dogs: range, 14–92 m	ClinPath (increased ALP, ALT, cholesterol)	increased ALP	I
Gaskil et al. 1999	PHB	78	40 %	28.8 %–50.6 %	median 4; range, 1–16.4 mg/kg PO SID	median, 17.6; range, 4–70 μg/ml	median, 12.5; range, 0.3–96	Endocrine (decreased total T4, free T4, increased TSH) Also, ClinPath abnormalities were reported, i.e. increased ALT, ALP, AST, γ-GT, fasting bile acids and cholesterol, but no further details are provided.	eythyroid sick syndrome	I
Muller et al. 2000	PHB	12	91.6 %	100 % or 50.5 %–99.5 %	mean, 5; range, 4.8–6.6 mg/kg PO BID	range, 20–40 μg/mL	7.1 m	Endocrine (decreased total T4, free T4, increased TSH, cholesterol and total T3), Neurological (sedation for the first 3 days) No significant PHB’s effect on either of the adrenal function tests	euthyroid sick syndrome	I
Muller et al. 2000	PHB	12	87.5 %	75.9 %–107.3 %	mean, 5; range, 4.8–6.6 mg/kg PO BID	range, 20–40 μg/ml	7.1 m	ClinPath (increased ALP, ALT, γ-GT, decreased albumin), Neurological (sedation for the first 3 days)	increased ALP, ALT, γ-GT	I
Kantrowitz et al. 1999	PHB	55	NA	NA	NA	median, 25.3; range, 8.0–74.3 μg/ml	median, 7 m; range, 1–120 m	Endocrine (decreased T4, increased TSH)	eythyroid sick syndrome	I
Chauvet et al. 1995	PHB	5	100 %	100 %	NA	range, 20–47 μg/ml	13 m	Endocrine (increased ACTH, altered ACTH stimulation and dexamethasone supression test), ClinPath (increased ALT, ALP, decreased albumin, cholesterol), PU, PD	increased ACTH, altered ACTH stimulation and dexamethasone supression test, increased ALT, ALP, decreased albumin, cholesterol	I
Balazs et al. 1978	PHB	4	100 %	100 %	40 mg/kg PO SID	NA	1.8 m	ClinPath (increased ALP)	increased ALP	I
Conning and Litchfield 1971	PHB	NA	NA	NA	NA	NA	NA	ClinPath (increased ALP)	increased ALP	I
Sturtevant et al. 1977	PHB	2	100 %	100 %	4.4 mg/kg PO TID	NA	1 m	ClinPath (Increased ALT, ALP)	increased ALT and ALP	I
Thrift et al. 2010	PHB	1	NA	NA	6.4 mg/kg PO BID	NA	2 m	ClinPath (anemia, increased ALT, ALP, AST)	idiosyncrasic anemia	I & II
Kube et al. 2006	PHB	1	NA	NA	Initially 5 mg/kg PO BID for 4 days, then 3 mg/kg PO BID	NA	2 m	Dyskinesia (twitching episodes)	NA	II
Steiner et al. 2008	PHB	118	14.4 %	8.1 %–20.7 %	NA	Unclear	NA	ClinPath (Increased cPLI)	NA	II
Gaskill et al. 2000	PHB	88	9 %	3.0 %–15.0 %	NA	range, 39–130 mol/L	16 m	GI (pancreatitis, increased amylase and/or lipase activities)	increased amylase and/or lipase activities	II
March et al. 2004	PHB	11	NA	NA	mean, 12.4+/−5.7; range, 3.8–19.8 mg/kg PO SID	mean, 43.5+/−15.1; range, 22.8–66 μg/ml	median, 6; range, 20.4–132 m	Dermatological (superficial necrolytic dermatitis)	NA	II
Weiss 2005	PHB	3	NA	NA	NA	NA	NA	Blood dyscrasias (bone marrow necrosis-myelofibrosis)	NA	II
Jacobs et al. 1998	PHB	2	NA	NA	Case 1: 2.2 mg/kg PO BID; Case 2: 4.4 mg/kg PO BID	NA	Case 1: 5 m; Case 2: 3 m	Blood dyscrasias (neutropenia, thrombocytopenia), ClinPath (increase ALP)	NA	II
Weiss et al. 2002	PHB	1	NA	NA	NA	NA	NA	Blood dyscrasias (myelofibrosis)	NA	II
Bevier et al. 2010	PHB	1	NA	NA	NA	NA	NA	Dermatological (superficial necrolytic dermatitis)	NA	II
Bersan et al. 2014	PHB	16	NA	NA	median, 3; mean, 2.75+/−0.43; range, 1.60–7.25 mg/kg PO BID	median, 19; mean, 22.4+/−5.5; range, 13.2–30.5 μg/ml	median, 69.5; mean, 72.1+/−sd 45.8; range, 14–157 m	Blood dyscrasias (anemia and/or thrombocytopenia and/or neutropenia and/or pancytopenia)	anemia, pancytopenia	II
Volk et al. 2008 (case series)	PHB (monotherapy prior to the addition of other AEDs)	8	NA	NA	NA but was within normal reference values	NA	Approximately 2–3 m	Blood dyscrasias (bone marrow suppression)	NA	II
Habock and Pakozdy 2012	PHB	37	22 %	16.8 %–57.2 %	NA	NA	>1 m	Blood dyscrasias (anemia and/or thrombocytopenia and/or neutropenia and/or pancytopenia)	NA	II
Von Klopmann et al. 2006	PHB	1	NA	NA	2 mg/kg PO BID	NA		Blood dyscrasias (pancytopenia)	NA	II
Bizzeti et al. 2006	PHB	7	14.4 %	−11.6 %−40.2 %	NA	NA	NA	Pancreatitis, ClinPath (Increased amylase, lipase, cPLI)	NA	II
Mathis et al. 2014	PHB	1	NA	NA	2.1 mg/kg PO BID	27.5 μg/dL	6 m	Blood dyscrasias (bone marrow supression)	NA	II
Daminet et al. 1999	PHB	9	0 %	0 %	Initially 1.8–3 for one week, then 2.7–4.5 mg/kg PO BID	range, 65–150 pmol/L	0.8 m	No adverse effects	NA	NA
Dyer et al. 1994	PHB	6	0 %	0 %	5 mg/kg PO BID	range, 18–37 μg/ml	2 m	No PHB’s effect on endogenous ACTH and ACTH stimulation test	NA	NA

*Abbreviations*: *AED(s)* anti-epileptic drug(s), *BID* bis in die (twice daily), *Chloraz* Chlorazepate, *CSF* cerebrospinal fluid, *CL* confidence level, *Gaba* Gabapentin, *IE* idiopathic epilepsy, *LEV* Levetiracetam, *m* month(s), *NA* Not Available, *PHB* phenobarbital, *PD* polydipsia, *PU* polyuria, *PP* polyphagia, *PBr* potassium bromide, *Prim* primidone, *PO* per os, *SID* semel in die (once daily), *TID* ter in die (three times daily), *TPM* topiramate, *w* week(s), *y* year(s)

**Table 2:** Details of number of dogs, 95 % CI affected cases, AED doses and serum levels, treatment period and adverse effects

Studies	AED	No of dogs treated	Prevalence	95 % CI affected cases	Doses of AEDs	Serum levels of AEDs	Treatment period	Body system affected and adverse effects	Most common adverse effects	Adverse effect type
Rundfeldt et sl. 2015	Imepitoin	127 Imepitoin high dose group: 66 Imepitoin low dose group: 61	Imepitoin high dose group: 86 % Imepitoin low dose group: 82 %	Imepitoin high dose group: 77.6 %–94.3 % Imepitoin low dose group: 72.3 %–91.6 %	Imepitoin high dose group: 30 mg/kg PO BID Imepitoin low dose group: 1 mg/kg PO BID	NA	1st phase: 3 m 2nd phase: 3 m	Neurological (hyperactivity, disorientation), musculoskeletal (unspecified), gastro-intestinal (unspecified), respiratory (unspecified), urogenital (unspecified), other systems (unspecified), general (unspecified)	Disorientation, hyperactivity	I
Tipold et al. 2014	Imepitoin	116	46.6 %	37.5 %–55.7 %	10–30 mg/kg PO BID	NA	5 m	Neurological (sedation, hyperactivity), GI (PP, diarrhoea), PU, PD, Renal/Urinary disorders, ClinPath (increased creatinine)	PP, PD, PU, sedation, hyperactivity	I
Tipold et al. 2014 (ELAS)	Imepitoin	32	NA	NA	30, 90 or 150 mg/kg PO BID (adverse effects occurred mainly in the higest doses, i.e. 3X and 5X the recommended dose)	NA	6 m	Neurological (loss of righting reflex, ataxia, intermittent tremors, decreased activity, nystagmus), GI (vomiting, hypersalivation, white material in the faeces), ClinPath (increased creatinine), Ophtalmological (lacrimation, eye dryness, eye discharges, relaxed nictitating membranes, eyelid closure)	NA (infrequent adverse effects)	I
Loscher et al. 2004, Rieck et al. 2006	Imepitoin as monotherapy (12 dogs) and imepitoin as an adjunct to PHB or Primidone (17 dogs)	29	58.6 %	40.7 %–76.5 %	Imepitoin: Initially 5 mg/kg PO BID for 1 week, then 10–30 mg/kg PO BID. PHB: 6–23 mg/kg PO SID. Primidone: 25–53 mg/kg PO SID	Imepitoin: mean, 4,000; range, 3400–7300 ng/ml (2 h after dosing) and mean, 650 ng/ml (12 h after dosing). PHB: range, 15–45 μg/ml (2 dogs with adverse effects had 56.6–58.9 lg/mL). Prim: NA	mean, 7.7 ± 0.7 m	Neurological (ataxia, sedation), GI (PP), ClinPath (increased ALT, ALP, GLDH)	PP	I
Löscher et al. 2004 (ELAS)	Imepitoin	1st experiment: 6 2nd experiment: 6	0 %	0 %	1st experiment: 5 mg/kg PO BID 2nd experiment: 40 mg/kg PO BID	1st experiment: range, 20–120 ng/ml 2nd experiment: range, 4800–7400 ng/ml	1st experiment: 1.2 m 2nd experiment: 1.2 m	1st experiment: none 2nd experiment: none but increase in body weight	NA	I
EMA report 2012 (US field trial)	Imepitoin	110	NA	NA	range, 10–30 mg/kg PO BID	NA	NA	Neurological (ataxia, hyperactivity, anxiety, disorientation), ClinPath (increased enzymes-unclear which) tachypnoea, PD	ataxia, hyperactivity, anxiety, PD, increased liver enzymes	I
EMA report 2012 (unpublished clinical trials: Tipold 2006; Heit 2011; de Vries 2011)	Imepitoin	NA	NA	NA	30 mg/kg PO BID (Unclear if other doses were also used)	NA	NA	Neurological (ataxia, decreased motor activity, disorientation, hyperactivity, decreased sight, increased sensitivity to sound), GI (vomiting, diarrhoea, polyphagia), Renal (increase creatinine)	ataxia, decreased motor activity, disorientation, hyperactivity, decreased sight, increased sensitivity to sound, vomiting, diarrhoea	I
EMA report (GLP toxicity study 1)	Imepitoin	32	0 %	Doses of 0, 31.6 mg/kg: 0 % Other doses: NA	Doses of 0, 31.6, 100 and 316 mg/kg/day PO	NA	1 m	Neurological (decreased motor activity), GI (hypersalivation, vomiting), ECG modifications No adverse effects in the recommended doses; adverse effects occurred only in the highest doses	NA	I
EMA report (GLP toxicity study 2)	Imepitoin	NA	NA	NA	Doses of 0, 31.6, 82.5 and 215 mg/kg/day PO	NA	3.2 m (followed by a 1.2 m recovery period)	Only vomiting occurred in the 0 and 31.6 mg/kg/day doses; adverse effects occurred only in the highest doses	NA	I

*Abbreviations*: *AED(s)* anti-epileptic drug(s), *BID* bis in die (twice daily), *Chloraz* chlorazepate, *CSF* cerebrospinal fluid, *CL* confidence level, *Gaba* Gabapentin, *IE* idiopathic epilepsy, *LEV* Levetiracetam, *m* month(s), *NA* Not Available, *PHB* phenobarbital, *PD* polydipsia, *PU* polyuria, *PP* polyphagia, *PBr*, potassium bromide, *Prim* primidone, *PO* per os, *SID* semel in die (once daily), *TID* ter in die (three times daily), *TPM* topiramate, *w* week(s), *y* year(s)

**Table 3:** Details of number of dogs, 95 % CI affected cases, AED doses and serum levels, treatment period and adverse effects

Studies	AED	No of dogs treated	Prevalence	95 % CI affected cases	Doses of AEDs	Serum levels of AEDs	Treatment period	Body system affected and adverse effects	Most common adverse effects	Adverse effect type
Boothe et al. 2012	PBr	23	78.5 %	61.7 %–95.3 %	mean, 30.6; range, 26–35 mg/kg PO BID	mean, 1.9 +/− 0.6; range, 0.9–3.3 mg/ml	approximately 6 m	Neurological (ataxia, hyperactivity, sedation), GI (vomiting, diarrhoea, PP), PU, PD	sedation, hyperactivity, ataxia, PD, PU	I
Pearce 1990	PBr as an adjunct to PHB	10	40 %	9.6 %–70.4 %	PBr: 22 mg/kg PO SID (dose increases occurred) PHB: median, 3.3; mean, 3.8 mg/kg PO BID (dose was reduced by a mean of 50 % in 7/10 dogs during the PBr treatment)	PBr: mean, 810; range, 500–1625 mg/l PHB: mean, 29.7; range, 17–45 ug/ml	median, 7; mean, 7.8 m	Neurological (ataxia, sedation, hyperactivity), PU, PD	ataxia, letargy, PU, PD	I
March et al. 2002	PBr	6	20 %	−12.0 %−52.0 %	30 mg⁄kg PO BID	median, 245; range, 178–269 mg/dL	3.9 m (adverse effects occurred after this period when dose adjustments occurred (NA))	Neurological (ataxia, paraparesis, hyperactivity)	ataxia, paraparesis	I
Rossmeisl et al. 2009	PBr as an adjunct to PHB and/or other AEDs	1298	2 %	1.2 %–2.8 %	PBr: 44.9+/−1.7 mg/kg PO SID PHB: 6.3+/−0.4 mg/kg PO SID	PBr: 3.7+/−0.3 mg/ml PHB: 31.4+/−1.2 μg/dl	NA	Neurological (sedation, ataxia, paraparesis, tetraparesis)	sedation, ataxia, paraparesis, tetraparesis	I
Dayrell-Hart B et al. 1996	PBr	238	10.9 %	6.9 %–14.9 %	NA	21 affected dogs had >2.3 mg/ml and 5 affected dogs had <0.5 mg/ml	NA	Neurological (ataxia, sedation)	ataxia, sedation	I
Podell and Fenner 1993	PBr as an adjunct to PHB and/or other AEDs	23	78 %	61.1 %–94.9 %	PBr: mean, 20.75; range, 13–40 PO BID PHB: NA	PBr: 161 mg/dl PHB: 37.8 mcg/ml	mean, 15; range, 4–33 m	Neurological (ataxia, sedation), GI (PP), ClinPath (increased serum chloride), PU, PD	PU, PD, PP, sedation	I
Chang et al. 2006	PBr (monotherapy or as an adjunct to PHB)	Monotherapry: 4 Adjunctive Therapy: 10	Monotherapry: 62.5 % Adjunctive Therapy: 95 %	Monotherapy: 15.0–110.0 % Adjunctive therapy: 81 %–109 %	NA	NA	median, 18; range, 3–72 m	Neurological (ataxia, hyperactivity), Dermatological (pruritus), GI (PP), The adjunctive therapy group had also PU, PD and vomiting/diarhoea	Ataxia, hyperactivity, pruritus, PP	I
Yohn et al. 1992	PBr as an adjunct to PHB	1	NA	NA	NA	2.7 mg/ml	1 m	Neurological (sedation, ataxia, paraparesis, anisocoria)	NA	I
Kantowitz et al. 1999	PBr (monotherapy or as an adjunct to PHB)	Monotherapry: 15 Adjunctive therapy: 8	NA	NA	NA	Monotherapy: median, 1985; range, 500–3419 mg/dL Adjunctive therapy: PBr: median, 1399; range, 584–2438 mg/dL. PHB: median, 22.4; range, 10.9–40 μg/ml	Monotherapy: median, 14.5; range, 3–37 m Adjunctive therapy: PBr: median, 5; range, 3–72 m. PHB: median, 22; range, 3–96 m	Monotherapy: Normal Adjunctive therapy: Endocrine (decreased total T4, free T4)	NA	I
Srivastava et al. 2013	PBr as an adjunct to PHB	6	100 %	100 %	PBr: 30 mg/kg PO SID PHB: Initially 2.5 mg/kg, then 5 mg/kg PO SID.	NA	mean, 11.50+/− 1.23; range, 8–15 m (on PHB). Then, PBr started and 3 m later a reduction of 50 % in the dose of PHB was performed. After 6 m, PHB was completely withdrawn.	Neurological (ataxia), GI (hepatoxicity, anorexia, PP), ClinPath (increased ALT, ALP, AST, bile acids), PU, PD (polyphagia, PU, PD appeared after 1–1.5 years of PBr therapy)	PU, PD, PP	I
Shaw et al. 1996	PBr as an adjunct to PHB	1	NA	NA	PBr: 20 mg/kg PO SID PHB: 3.75 mg/kg PO BID	PBr: 1100 mg/l. PHB: 20.4 μg/ml	Approximately 21 m	After PBr initiation: Neurological (sedation, ataxia)	NA	I
Paull et al. 2003	PBr	5	60 %	17.1 %–102.9 %	Initially 100 mg/kg PO BID for 2 days. Then, 30 mg/kg PO SID for 180 days.	range, 88–300 mg/dL (only one dog was >300 mg/dl)	6 m	Endocrine (Euthyroid sick syndrome with decreased TT4 and normal TSH) Placebo group had the same results	NA	I
Stabile et al. 2014	PBr as an adjunct to PHB	1	NA	NA	PBr: Initially, 400 mg/kg divided in six daily doses for four days. Then, 14 mg/kg PO BID PHB: Initially, 2.7 mg/kg, then 5 mg/kg and finally 6.4 mg/kg PO BID.	PBr: 15.9 mg/ml; PHB: 23.7 μg/ml	≥26 m	Neurological (sedation, ataxia, generalised appendicular repetitive myoclonus), ClinPath (pseudohyperchlormia, increased ALP)	NA	I & II
Gaskill and Kimber 2010	PBr	32	85.9 %	73.8 %–98.0 %	NA	NA	12 m	Neurological (ataxia, sedation, hyperactivity, aggression), GI (PP, anorexia, vomiting, diarrhoea, pancreatitis), Dermatological (skin problems), ClinPath (increased ALP, ALT, amylase, lipase), PU, PD	vomiting, sedation, PP, PU, PD	I & II
Volk et al. 2008	PBr as an adjunct to PHB (prior to addition of other AEDs)	14	100 %	100 %	PBr and PHB: NA but were within normal reference values	PBr: 1.7+/−0.4 mg/ml PHB: 35.5+/−6.3 μg/ml	≥2–6 m	Neurological (ataxia, aggression), GI (PP, vomiting, pancreatitis), ClinPath (increased ALT, ALP) PU, PD	increased ALT, ALP, ataxia, aggression	I & II
Gaskill et al. 2000	PBr as an adjunct to PHB	Clinical trial: 6 Case series: 19	Clinical trial: 50 % Case series: 37 %	Clinical trial: 10.0 %–90.0 % Case series: 15.3 %–58.7 % (pancreatitis); 47.5 %–89.3 % (increased enzymes only)	Clinical trial: NA Case series: NA	Clinical trial: NA Case series: PBr: range, 12.5-37.5 mmol/L; PHB: range, 54–190 imol/L	Clinical trial: approximately 1 year Case series: NA	GI (pancreatitis, increased amylase and/or lipase activities)	pancreatitis, increased amylase and/or lipase activities	II
Steinmetz et al. 2012	PBr as an adjunct to PHB	1	NA	NA	101.19 mg/kg SID PO (added at the beginning of the 4th year) PHB: 4.9 mg/kg BID PO	PBr: 45 mmol/l PHB: 168.52 μmol/l	48 m (adverse effect occurred after the 48 m)	Neurological (neuromyopathy with generalised low motor signs)	NA	II
Mackay and Mitchell 1998	PBr as an adjunct to PHB	1	NA	NA	PBr: Initially 200 mg/kg PO BID for 3 days, then 30 mg/kg PO SID PHB: 5 mg/kg PO BID.	PBr: NA. PHB: 126 umol/L	3 d (signs started 3 d after the loading dose of PBr was initiated)	ClinPath (artifactual hyperchloraemia and negative anion gap), Neurological (pacing, disorientation), GI (vomiting) The Neurological and GI signs were attributed to hypercloraemia	NA	II
Boynosky and Stokking 2014	PBr as an adjunct to PHB	2	NA	NA	Initially 40 mg/kg PO SID, then 60 mg/kg PO SID (case 1) or 86 mg/kg PO SID (case 2)	Case 1: 2.9 mg/mL; Case 2: initially 0.8, then 3 mg/ml (after 7.5 months of treatment)	12 m [adverse effects occured 3 (case 1) and 8 (case 2) m after the dose increase]	Dermatological (panicculitis) accompanied by sedation and anorexia	NA	II
Steiner et al. 2008	PBr (monotherapy or as an adjunct to PHB)	Monotherapy: 98 Adjunctive therapy: 121	14 %	Monotherapy: 8.2 %–22.4 % Adjunctive: 5.9 %–17.3 %	NA	range, 0.5–4.2 mg/ml (majority of dogs; range, 1–2 mg/ml)	NA	ClinPath (increased cPLI)	NA	II
Bizzeti et al. 2006	PBr as an adjunct to PHB	7	43 %	6.1 %–79.4 %	NA	NA	NA	Pancreatitis, ClinPath (increased amylase, lipase,cPLI)	NA	II

*Abbreviations*: *AED(s)* anti-epileptic drug(s), *BID* bis in die (twice daily), *Chloraz* Chlorazepate, *CSF* cerebrospinal fluid, *CL* confidence level, *Gaba* Gabapentin, *IE* idiopathic epilepsy, *LEV* Levetiracetam, *m* month(s), *NA* Not Available, *PHB* phenobarbital, *PD* polydipsia, *PU* polyuria, *PP* polyphagia, *PBr* potassium bromide, *Prim* primidone, *PO* per os, *SID* semel in die (once daily), *TID* ter in die (three times daily); *TPM* Topiramate; *w* week(s), *y* year(s)

**Table 4:** Details of number of dogs, 95 % CI affected cases, AED doses and serum levels, treatment period and adverse effects

Studies	AED	No of Dogs	Prevalence	95 % CI affected case	Doses of AEDs	Serum levels of AEDs	Treatment period	Body system affected and adverse effects	Most common adverse effects	Adverse effect type
Volk et al. 2008	LEV as an adjunct to PHB and/or PBr	14	7.14 %	−6.3 %−20.6 %	LEV: 10 mg/kg for 2 m, 20 mg/kg for further 2 m, 10–20 mg/kg until 6 m and then 10–20 mg/kg long-term PO TID PHB and PBr: NA but were within normal reference values	PHB: 35.5+/−6.3 μg/ml, PBr:1.7+/−0.4 mg/ml, (prior LEV initiation and 2 m after initiation).	≥2–6 m	Neurological (sedation)	sedation	I
Volk et al. 2008 (case series)	LEV as an adjunct to PHB and/or PBr and/or gaba and/or TPM	8	25 %	−2.6 %–18.6 %	LEV: 30–32 mg/kg PO TID TID PHB and PBr: NA but were within normal reference values	NA	Approximately 2–3 m	Neurological (sedation)	sedation	I
Muñana et al. 2012	LEV as an adjunct to PHB and/or PBr and/or gaba and/or zonisamide	28	57 %	38.7 %–75.3 %	LEV: median, 20.6; range, 17–23.1 PO TID PHB: median, 7.2; range, 3.8–17.2 mg/kg PO SID. PBr: median, 34.0; range, 13.6–84.2 mg/kg PO SID	LEV: range, <2-50.8 μg/mL. PHB: mean, 28.13; range, 15.77–36.40 μg/mL PBr: mean, 186.20; range, 71.18–390 mg/dL	9 m (during the 5th m no AED was administered)	Neurological (ataxia, hyperactivity), GI: (anorexia, vomiting)	ataxia	I
Steinberg 2004	LEV as an adjunct to PHB and PBr	15	0 %	0 %	LEV: range, 7.1–23.8 mg/kg PO TID PB and PBr: NA	PHB: mean, 32.1+/−14.4 μg/ml. LEV: NA PBr: 2.2+/−0.7 mg/dl	median, 38; range, 13.8–95.5 m	No adverse effects attributed to LEV	NA	I
Packer et al. 2015	LEV as an adjunct to PHB and PBr	52	46 %	32.5 %–59.6 %	Maintenance group: mean, 19.5; range, 9–26 mg/kg PO TID. Pulse group: Initial dose at 60 mg/kg followed by 20 mg/kg PO TID	NA	Maintenance group: mean, 1.4; range, 0.3–7.5 y Pulse group: mean, 0.8; range, 0.3–3.4 y	Neurological (ataxia, sedation, aggression, hyperactivity), GI (PP, vomiting, diarrhoea), PD (Three times more often in the pulse group)	ataxia, sedation	I
Fredso et al. 2015	LEV	6	84 %	53.8 %–113.2 %	median, 31; mean, 30.4; range, 27.6–51.5 PO TID	median, 114; mean, 93; range, 18–137 μmol/L	2–12 m	Neurological (ataxia, sedation, hyperactivity, disobedience, attention seeking), GI (PP, anorexia, vomiting), PU, PD	PP	I
Moore et al. 2010	LEV	6	16.6 %	–13.2 %–46.4 %	At day one, a single dose was administered: mean, 21.7; range, 20.8–22.7 mg/kg PO. Then: range, 20.8–22.7 mg/kg PO TID for 6 d	289.31+/−51.68 μg/mL	0.25 m	GI (vomiting) (only one episode at the first d)	NA	I

*Abbreviaions*: *AED(s)* anti-epileptic drug(s), *BID* bis in die (twice daily), *Chloraz* Chlorazepate, *CSF* cerebrospinal fluid, *CL* confidence level, *Gaba* Gabapentin, *IE* idiopathic epilepsy, *LEV* Levetiracetam, *m* month(s), *NA* Not Available, *PHB* phenobarbital, *PD* polydipsia, *PU* polyuria, *PP* polyphagia, *PBr* potassium bromide, *Prim* primidone, *PO* per os, *SID* semel in die (once daily), *TID* ter in die (three times daily), *TPM* Topiramate, *w* week(s), *y* year(s)

**Table 5:** Details of number of dogs, 95 % CI affected cases, AED doses and serum levels, treatment period and adverse effects

Study	AED	No of dogs	Prevalence	95 % CI affected case	Doses of AEDs	Serum levels of AEDs	Treatmentperiod	Body system affected and adverse effects	Most common adverse effects	Adverse effect type
von Klopmann et al. 2007	Zonisamide as an adjunct to PHB and/or PBr	11	72.7 %	46.4 %–99.0 %	Zonisamide: mean, 8.9; range, 5–11 mg/kg PO BID PHB and PBr: NA but continued unchanged or reduced if appropriate	Zonisamide: median, 19.2; range, 15.2–38. 4 lg/ml. PHB: median, 121; range, 66–150 5 lmol/l. PBr: median 1.2; range, 0.7–1.7 g/l.	range, 4–17 m	Neurological (ataxia, sedation), ClinPath (increased ALP, ALT and GLDH)	ataxia, sedation, increased ALP	I
Chung et al. 2012	Zonisamide	10	10 %	−8.6 %–28.6 %	median 9.5; mean 8.65; range 2.5–12 mg/kg PO BID	range, 15.24–22.41 mg/mL	median, 12; mean, 11.2 m	Neurological (sedation), GI (vomiting, anorexia)	sedation, vomiting, anorexia	I
Dewey et al. 2004	Zonisamide as an adjunct to PHB and/or PBr and/or felbamate and/or gaba and/or cloraz	12	50 %	21.7 %–78.3 %	Zonisamide: mean, 8.9; range, 5–11 mg/kg PO BID. Other AEDs: NA but in 9/12 dogs concurrent AEDs doses were eliminated or reduced.	Zonisamide: median, 23.5; mean, 21.2 μg/mL.	mean, 8; median, 9; range, 2–18 m	Neurological (ataxia, sedation), GI (vomiting), ClinPath (ALP)	ataxia, increased ALP	I
Walker et al. 1988	Zonisamide	40	NA	NA	10, 30 or 75 mg/kg PO SID	range, 10–140 ug/ml	13 m	Neurological (aggression) GI (emaciation), ClinPath (increased ALP, decreased albumin)	aggression, increased ALP, decreased albumin	I
Boothe et al. 2008	Zonisamide	8	0 %	0 %	6.9 mg/kg IV SID or 10.3 mg⁄kg PO SID	range, 6–55 mcg⁄ml	2 m	Endocrine (Decreased total T4) (However, total T4 was only slightly decreased at the study end)	NA	I
Cook et al. 2011	Zonisamide	1	NA	NA	range, 7.9–8.4 mg/kg PO BID	38 μg/mL	18 m	ClinPath (mixed acid base disorder)	NA	II
Miller et al. 2011	Zonisamide	1	NA	NA	7.7 mg/kg PO BID	NA	0.3 m	GI (hepatoxicity)	NA	II

*Abbreviations*: *AED(s)* anti-epileptic drug(s), *BID* bis in die (twice daily), *Chloraz* Chlorazepate, *CSF* cerebrospinal fluid, *CL* confidence level, *Gaba* Gabapentin, *IE* idiopathic epilepsy, *LEV* Levetiracetam, *m* month(s), *NA* Not Available, *PHB* phenobarbital, *PD* polydipsia, *PU* polyuria, *PP* polyphagia, *PBr* potassium bromide, *Prim* primidone, *PO* per os, *SID* semel in die (once daily), *TID* ter in die (three times daily), *TPM* Topiramate, *w* week(s), *y* year(s)

**Table 6:** Details of number of dogs, 95 % CI affected cases, AED doses and serum levels, treatment period and adverse effects

Studies	AED	No of dogs treated	Prevalence	95 % CI affected cases	Doses of AEDs	Serum levels of AEDs	Treatment period	Body system affected and adverse effects	Most common adverse effects	Adverse effect type
Schwartz-Porsche et al. 1982	Prim	30	NA	NA	range, 13–100 mg/kg PO SID	range, 6–37 μg/ml	range 6–96 m	Neurological (sedation, hind limb weakness), GI (PP), PU, PD, ClinPath (ALP, SGPT)	sedation, PU, PD	I
Schwartz-Porsche et al. 1985	Prim	20	NA	NA	range, 17–107 mg/kg PO SID	range, 0.5–58 μg/ml	mean, 14; range, 6.0–35 m	Neurological (sedation, hind limb weakness, ataxia), GI (PP), PD, ClinPath (ALT, ALP, GLDH)	sedation, PU, increased ALT, ALP and GLDH	I
Farnbach et al. 1984	Prim	23	4.3 %	−4.0 %–12.6 %	range, 15.2–82 mg/kg PO SID	range, 4.8–70.7 μg/ml	NA	Neurological (sedation, ataxia)	sedation, ataxia	I
Cunningham et al. 1983	Prim	15	NA	NA	10.6–39.4 mg/kg PO TID	mean, 2.4 μg/ml	9 m	Neurological (disorientation, ataxia, hyperactivity, pacing), GI (PP), PU, PD	PU, PD, PP, drowsiness, ataxia	I
Poffenbarger et al. 1985	Prim (monotherapy or as an adjunct to PHB)	3	NA	NA	Varied	NA	8–84 m	GI (chronic hepatopathy/toxicity-hepatic chirosis)	chronic hepatopathy/toxicity	I
Bunch et al. 1987	Prim	1	NA	NA	13 mg/kg PO BID	NA	4 d	Neurological (hyperactivity)	NA	I
Bunch et al. 1984	Prim	22	93 %	82.3 %–103.6 %	Prim: 33+/−19 mg/kg PO SID Other AEDs: NA	NA	range, 6–120 m	GI (chronic hepatopathy/toxicity), ClinPath (increased ALP, ALT, AST, γ-GT, bile acids)	chronic hepatoxicity, increased ALP, ALT, AST, bile acids	I
Bunch et al. 1982	Prim	2	NA	NA	Case 1: 750 mg in total PO BID Case 2: 250 mg in total PO BID	NA	24 m	GI (chronic hepatopathy/toxicity)	chronic hepatoxicity	I
EPAR (US field trial)	Prim	110	NA	NA	NA	NA	NA	Neurological (ataxia, hyperactivity, anxiety, disorientation), ClinPath (increased enzymes-unclear which) tachypnoea, PD	ataxia, PD, increased liver enzymes	I
Raw and Gaskell 1985	Prim	52	35 %	22.0 %–48.0 %	NA	NA	48 m	Neurological (ataxia), GI (PP), PD	ataxia, PD, PP	
Meyer and Noonan 1981	Prim	6	100 %	100 %	30–40 mg/kg PO BID	NA	3 m	ClinPath (increased ALP, ALT)	increased ALT, ALP	I
Sturtevant et al. 1977	Prim	2	100 %	100 %	17.6 mg/kg POTID	NA	1 m	ClinPath (anemia, increased ALP)	increased ALP	I & II
Jacobs et al. 1998	Prim	1	NA	NA	25 mg/kg PO BID	NA	2 m	Blood dyscrasias (neutropenia, anemia, thrombocytopenia), ClinPath (decreased albumin, increased ALP)	NA	II
Henricks 1987	Prim	1	NA	NA	62 mg in total PO BID	NA	2 m	Dermatitis	NA	II
Balazs et al. 1978	Prim	4	0 %	0 %	40–80 mg/kg PO SID	NA	1.75 m	No adverse effects	NA	NA
Bunch et al. 1985	Prim	6	0 %	0 %	NA	NA	NA	No adverse effects	NA	NA

*Abbreviations*: *AED(s)* anti-epileptic drug(s), *BID* bis in die (twice daily), *Chloraz* Chlorazepate, *CSF* cerebrospinal fluid, *CL* confidence level, *Gaba* Gabapentin, *IE* idiopathic epilepsy, *LEV* Levetiracetam, *m* month(s), *NA* Not Available, *PHB* phenobarbital, *PD* polydipsia, *PU* polyuria, *PP* polyphagia, *PBr* potassium bromide, *Prim* primidone, *PO* per os, *SID* semel in die (once daily), *TID* ter in die (three times daily), *TPM* Topiramate, *w* week(s), *y* year(s)

**Table 7:** Details of number of dogs, 95 % CI affected cases, AED doses and serum levels, treatment period and adverse effects

Studies	AED	No of dogs	Prevalence	95 % CI affected cases	Doses of AEDs	Serum levels of AEDs	Treatment period	Body system affected and adverse effects	Most common adverse effects	Adverse effect type
Govendir et al. 2005	Gaba as an adjunct to PHB and/or PBr	17	76.5 %	56.3 %–96.6 %	Gaba: median, 35; range, 32–40 mg/kg PO SID. PHB: median, 8; range, 6–26 mg/kg PO SID. PBr: median, 24; range, 14–56 mg/kg PO SID.	Gabapentin: NA PHB and PBr: within normal reference values	4 m	Neurological (sedation, ataxia), GI (PP, pancreatitis, chronic hepatoxicity), ClinPath (increased ALP, triglycerides), PU, PD	ataxia, sedation	I
Platt et al. 2006	Gaba as an adjunct to PHB and PBr	11	54.5 %	25.1 %–83.9 %	mean, 10.9; range, 9.3–13.6 mg/kg PO TID	median, 6.8; mean, 8.4; range, 2.2–20.7 mg/l	3 m	Neurological (ataxia, sedation)	ataxia, sedation	I
Dewey et al. 2009	Pregabalin as an adjunct to PHB and PBr	11	91 %	74.1 %–107.9 %	Pregabalin: 2 mg/kg PO TID. The dose was increased by 1 mg/kg PO TID each w until 3 or 4 mg/kg PO TID. PHB and PBr: NA but were within normal reference values	Pregabalin: median, 7.3; mean, 6.4; range 2–11 μg/ml PHB: median, 27.1; mean, 27.7; range 19.8–40 μg/ml PBr: median, 1,6; mean, 1.9; range, 0.2–2.81 mg/ml	3 m	Neurological (ataxia, sedation), ClinPath (increased ALP, ALT) PU, PD, PP were also recorded but were associated to the combination therapy with PHB and PBr	ataxia, sedation	I
Ruehlmann et al. 2001	Felbamate as an adjunct to PHB	6	33.3 %	−4.4 %–71.0 %	Felbamate: median, 63 (initial dose) and 77 (final dose); range, 62–220 mg/kg PO SID. PHB: 3.75 mg/kg PO BID (discontinued 2 m after felbamate initiation)	median, 35; mean, 13–55 mg/l	median, 9 m	Haematological (leucopenia, lymphopenia, thrombocytopenia), keratoconjunctivitis sicca	leucopenia, lymphopenia, thrombocytopenia	I
McGee et al. 1998	Felbamate	NA	NA	NA	Sub-chronic group: 250, 500, and 1000 mg/kg PO SID Chronic group: 100 and 300 mg/kg PO SID	range, 16.5–79 μg/ml	Sub-chronic group: 3 m chronic group: 12 m	Sub-chronic group: Neurological (ataxia, sedation, tremors), GI (vomiting, salivation), ClinPath (increased ALT) Chronic group: Neurological (ataxia, limb rigidity, tremors), GI (vomiting, salivation), ClinPath (increased ALT, ALP)	Sub-chronic group: ataxia, sedation, tremors, vomiting, salivation, increased ALT. Chronic group: ataxia, limb rigidity, convulsions, vomiting, salivation, increased ALT, ALP.	I
Dayrell-Hart et al. 1996	Felbamate as an adjunct to PHB and PBr	16	25 %	3.8 %–46.2 %	NA	NA	NA	GI (chronic hepatotoxicity)	chronic hepatotoxicity	I
Bunch et al. 1985	Phenytoin (monotherapy or as an adjunct to Prim)	Monotherapy: 8 Adjunctive therapy: 8	Monotherapy: 0 % Adjunctive therapy: 37 %	Monotherapy: 0 % Adjunct therapy:−1 %–16.7 %	NA	NA	NA	Monotherapy: None Adjunctive therapy: GI (anorexia, emaciation), ClinPath (increased ALP, ALT, bilirubin, bile acids, γ-GT, decreased albumin) These dogs were eventually euthanised.	Monotherapy: NA Adjunctive therapy: anorexia, increased ALP, ALT, bilirubin, bile acids, γ-GT, decreased albumin	I
Bunch et al. 1984	Phenytoin (monotherapy or as an adjunct to other AED(s))	Monotherapy: 7 Adjunctive therapy: 19	NA	ΝΑ	Phenytoin: mean, 21+/− 11 mg/kg PO SID Prim: mean, 33+/−19 mg/kg PO SID. Other AEDs: NA	NA	range, 6–120 m	GI (chronic hepatoxicity), ClinPath (increased ALP, ALT, AST, bile acids)	Chronic hepatoxicity, increased ALP, ALT, AST, bile acids	I
Meyer and Noonan 1981	Phenytoin	6	100 %	100 %	13–19 mg/kg PO TID	NA	3 m	ClinPath (increased ALP, ALT)	increased ALP and ALT	I
Sturtevant et al. 1977	Phenytoin	2	100 %	100 %	22 mg/kg PO TID	NA	1 m	ClinPath (increased ALP, ALT)	increased ALP and ALT	I
Bunch et al. 1982	Phenytoin as an adjunct to Prim	3	NA	NA	Case 1: Prim: 250 mg PO BID; Phenytoin: NA. Case 2: Prim: 750 mg PO BID Phenytoin: 100–233 mg PO TID Case 3: Prim: NA PHB: 150 mg PO SID Phenytoin: 750 mg PO SID 1000 mg PO SID	NA	Case 1: 48 m. Case 2: 30 m Case 3: 36 m	GI (chronic hepatoxicity)	chronic hepatoxicity	I
Weiss et al. 2002	Phenytoin	1	NA	NA	NA	NA	NA	Blood dyscrasias (myelofibrosis)	NA	II
Bunch et al. 1987	Phenytoin as an adjunct to PHB and/or Prim	3	NA	NA	Case 1: Phenytoin: 5 mg/kg PO BID, then increased up to 15 mg/kg PO TID. PHB: 0.8 mg/kg PO BID, then increased up to 13 mg/kg PO BID Case 2: Phenytoin: 7.5 mg/kg PO BID, then increased up to 15 mg/kg PO TID. PHB: 1.1 mg/kg PO BID, then increased up to 4.5 mg/kg PO TID. Prim: 18.5 mg/kg PO TID Case 3: Phenytoin: 5 mg/kg PO SID, then increased up to 21 mg/kg PO BID. PHB: 3 mg/kg PO BID. Prim: 13 mg/kg PO SID, then increased up to 26 mg/kg PO BID	Case 1: NA Case 2: NA Case 3: NA	Case 1: 27 months Case 2: 15 months Case 3: 8 months	GI (hepatotoxicity)	NA	II
Nash et al. 1977	Phenytoin	1	NA	NA	100 mg in total	NA	1 d	Idiosyncrasic hepatitis	NA	II
Bunch et al. 1990	Phenytoin	8	0 %	0 %	40 mg/kg PO TID	NA	13.5 m	No adverse effects	NA	NA
Nafe 1981	Valproate (monotherapy or as an adjunct to PHB and/or Prim and/or phenytoin)	Monotherapy: NA Adjunctive therapy: 57	Monotherapy: NA Adjunctive therapy: 2 %	Monotherapy: NA Adjunctive therapy:−1.7 %–5.1 %	Sodium Valproate: Monotherapy: 200 mg/kg. Adjunctive therapy: range, 25–40 mg/kg PO SID. PHB, Prim and Phenytoin: NA.	NA	mean, 4.9; range, 1–8 m	Neurological (ataxia, sedation), dermatological (alopecia), GI (vomiting)	Sedation, alopecia	I
Kiviranta et al. 2013	TPM	10	NA	NA	TPM: Initially 2 mg/kg PO BID for 0.5 m, then 5 mg/kg PO BID for 2 m, and then 10 mg/kg PO BID for 2 m and then 10 PO TID for 2 m. PHB, PBr and LEV: NA but were within normal reference values	NA	2–6 m	Neurological (sedation, ataxia), ClinPath (increased ALP, ALT), weight lose	sedation, ataxia, increased ALP, ALT	I

*Abbreviations*: *AED(s)* anti-epileptic drug(s), *BID* bis in die (twice daily), *Chloraz* Chlorazepate, *CSF* cerebrospinal fluid, *CL* confidence level, *Gaba* Gabapentin, *IE* idiopathic epilepsy, *LEV* Levetiracetam, *m* month(s), *NA* Not Available, *PHB* phenobarbital, *PD* polydipsia, *PU* polyuria, *PP* polyphagia, *PBr* potassium bromide, *Prim* primidone, *PO* per os, *SID* semel in die (once daily), *TID* ter in die (three times daily), *TPM* Topiramate, *w* week(s), *y* year(s)

### Phenobarbital

There was an overall strong level of evidence provided for the phenobarbital safety profile. Forty-three studies [3, 23, 25, 27–29, 31, 33, 36–40, 42, 43, 45, 46, 49, 50, 52, 53, 60, 69, 71, 74–76, 79–81, 83–85, 87, 93–95, 98, 100, 103, 107, 109, 111] presented data about the safety profile of phenobarbital as a monotherapy agent, giving a combined sample size of 1003 dogs.

Twenty-seven studies reported type I adverse effects (dose dependent/predictable), including neurological signs and clinical pathological findings as the most common (Table 1). Specifically, these adverse effects most commonly included increased serum ALP activity and ALT activity followed by sedation, ataxia, polydipsia, polyuria, polyphagia, euthyroid sick syndrome, hyperactivity, increased serum γ-GT activity, decreased serum albumin and diarrhoea. Less commonly, vomiting, pruritus, chronic clinical hepatopathy/toxicity, increased serum GLDH activity, AST activity, cholesterol, bile acids and bilirubin activity, aggression, anorexia and decreased serum total proteins were reported (Fig. 2). Twenty studies reported alterations in one or multiple liver enzymes, but only three of them reported chronic clinical hepatopathy/toxicity. The occurrence of euthyroid sick syndrome and asymptomatic/subclinical pancreatitis may have been underestimated because only a very few studies included the relevant diagnostic tests to evaluate these disorders. Two studies [31, 33] evaluated the effect of phenobarbital on adrenal function and found no significant effect. The same studies reported no adverse effects, although they focused on reporting adverse effects related to adrenal function. One study [71] reported that adrenal function might have been affected by phenobarbital (i.e. altered ACTH stimulation and dexamethasone suppression tests despite normal endogenous ACTH concentrations).

Fifteen studies reported type II adverse effects (idiosyncratic/unpredictable) with hematological signs as the most common (Table 1). Specifically, the most commonly reported adverse effects included blood dyscrasias (i.e. anemia, thrombocytopenia, leucopenia and/or pancytopenia) followed by pancreatitis, superficial necrolytic dermatitis and lastly dyskinesia (i.e. twitching episodes) (Fig. 3). In one study [50] elevated serum canine pancreatic lipase immunoreactivity (cPLI) concentration was detected in some dogs, but further diagnostic tests and clinical evaluation was not performed; thus, it is unknown if the dogs had developed clinical pancreatitis. In addition, in one study [81], 212 dogs with IE treated with phenobarbital monotherapy or combined therapy were evaluated and only 9 were found to have phenobarbital-induced blood dyscrasias, giving a prevalence of 4.2 % (95 % CI: 1.5–7.0 %).

Adequate data to allow calculations of the prevalence of adverse effects was reported in 25/43 (58 %) of the studies (Table 1). From these, 13/25 (52 %) showed >50 % prevalence of at least one adverse effect for the specific period of treatment they were conducted. Based on the 95 % CI, the majority of the study populations experienced at least one adverse effect in 11/25 (44 %) studies (Table 1).

Adequate information about the treatment period was reported in 34/43 (79 %) (Table 1). From these, in 10/34 (29 %) the treatment period was relatively short (<6 months). Adequate information about the dose was provided in 28/43 (65 %) studies (Table 1). From these, 19/28 (68 %) and 9/28 (32 %) reported type I and type II adverse effects respectively. The maintenance doses were higher than the recommended dose range in type I adverse effects (11/19, 58 %), while within normal ranges for type II adverse effects (7/9, 78 %). Adequate information about the phenobarbital serum levels was reported in 24/43 (56 %) studies (Table 1). From these 17/24 (71 %) and 4/24 (17 %) reported doses for type I and type II adverse effects respectively. The phenobarbital serum levels were higher than the recommended therapeutic ranges in type I adverse effects (9/17, 53 %), while within normal ranges for type II adverse effects (4/4, 100 %).

### Imepitoin

There was an overall strong level of evidence provided for the imepitoin safety profile. Six studies [25, 26, 44, 48, 112, 113] presented data about the safety profile of oral imepitoin either as monotherapy (all studies) and/or an adjunct to other AEDs (two studies) [44, 48], giving a combined sample size of 458 dogs. Two studies [25, 44] included both a clinical trial and ELAS part. EMA imepitoin assessment report [112] included two unpublished GLP toxicity studies and three unpublished clinical trials [117–119]; the latter three studies were not clearly distinguished in the EMA report and thus they were considered as one study.

Nine studies reported type I adverse effects including neurological and gastro-intestinal signs as the most common (Table 2). Specifically, the most common adverse events included ataxia and polyphagia, followed by sedation, hyperactivity, increased serum creatinine activity, vomiting and diarrhoea, disorientation and polydipsia. Less commonly, polyuria, anxiety, tachypnea, hypersalivation, decrease in sight and motor activity, prolapsed nictitating membrane and increased sensitivity to sound were reported (Fig. 4).

It is worth mentioning that the two GLP toxicity studies [112] and the ELAS part of one study [25] reported a few further adverse effects. However, these specific adverse effects were not included in the assessment above (see Table 2 for details) because higher than therapeutic doses were administered (3X, 5X or higher the recommended dose) which intended to evaluate the potential toxicity and tolerability of the drug. The same studies showed no adverse effects when imepitoin was administered in the recommended doses (≤30 mg/kg) apart from vomiting, hypersalivation and prolapsed nictitating membrane (as described above).

Adequate data to allow calculations of the prevalence of adverse effects was reported in 5/9 (55 %) of the studies (Table 2). From these, only two studies showed >50 % prevalence of adverse effects for the specific treatment periods within which they were conducted. However, one of the studies included a group of dogs receiving imepitoin adjunctive therapy to phenobarbital or primidone and therefore the prevalence may have been overestimated in these. Based on the 95 % CIs, the majority of the combined study populations experienced adverse effects in only one study (Table 2).

Adequate information about the treatment period was reported in 7/9 (78 %) (Table 2). From these, in 6/7 (86 %) the treatment period was relatively short (<6 months). Adequate information about the doses was provided in all studies (Table 2). The imepitoin maintenance doses were mainly at the higher recommended dose, but the toxicology studies in which higher than recommended doses were used. Adequate information about the imepitoin serum levels was reported in 2/9 (22 %) studies (Table 2), but no correlation was detected between imepitoin serum levels and adverse effects.

### Potassium bromide

There was an overall strong level of evidence provided for potassium bromide safety profile as monotherapy and weak as an adjunctive therapy. Twenty-one studies [3, 23, 29, 39, 51, 53, 66, 67, 73, 75, 77, 86, 88, 93, 96, 101, 102, 106, 110, 116] presented data about the safety of potassium bromide either as monotherapy (8 studies) [3, 23, 29, 39, 50, 51, 73, 86] or adjunctive therapy to phenobarbital and/or other AEDs (16 studies) [3, 39, 50, 53, 66, 67, 75, 77, 88, 93, 96, 101, 102, 106, 110, 116], giving a combined sample size of 1940 dogs.

Fifteen studies reported type I adverse effects, including neurological signs as the most common (Table 3). From these, 7/15 (47 %) and 10/15 (67 %) studies reported type I adverse effects for potassium bromide monotherapy and adjunctive therapy, respectively. Specifically, in all the studies, the adverse effects most commonly reported were ataxia followed by sedation, polyuria, polydipsia, polyphagia, paraparesis, hyperactivity, vomiting, increased serum ALP and ALT activity. Less commonly, diarrhoea, anorexia, aggression and then tetraparesis, skin issues, euthyroid sick syndrome, anisocoria, chronic clinical hepatopathy/toxicity, and lastly increased serum chloride, bile acids and AST activity were reported. A similar pattern was shown in the adjunctive therapy studies; diarrhoea, skin conditions and increased amylase and lipase were not reported. In the monotherapy studies, the most common adverse effects were ataxia, sedation and hyperactivity followed by polyuria, polydipsia, polyphagia and diarrhoea; clinical hepatopathy/toxicity, tetraparesis, anisocora, euthyroid sick syndrome, increased serum bile acids and AST activity were not reported (Fig. 5). In one study [51], euthyroid sick syndrome was detected in both potassium bromide-treated and placebo group, indicating that this AED might not affect thyroid function. In another study [39], potassium bromide monotherapy did not affect the thyroxin serum levels.

Nine studies reported type II adverse effects, including gastro-intestinal signs as the most common (Table 3). Specifically, pancreatitis was most commonly reported, followed by panniculitis, generalized appendicular repetitive myoclonus, neuromyopathy with generalized lower motor signs and hyperchloraemia with negative anion gap (Fig. 6). The latter was detected in a dog, two days after a loading dose (200 mg/kg BID orally) of potassium bromide was given, but not after regular maintenance doses. In one study [50], although clinical pancreatitis due to potassium bromide or the phenobarbital/potassium bromide combination was suspected, it was not confirmed as the history and clinical status of the dogs were unknown.

Adequate data to allow calculations of the prevalence of adverse effects was reported in 14/21 (67 %) of the studies (Table 3). From these, 7/15 (47 %) showed >50 % prevalence of adverse effect for the specific period of treatment they were conducted. Based on the 95 % CI, the majority of the combined study population experienced at least one adverse effect in 7/15 (47 %) studies (Table 3).

Adequate information about the treatment period was reported in 17/21 (81 %) (Table 3). From these, in 5/17 (29 %) the treatment period was relatively short (<6 months). Adequate information about the doses was provided in 13/21 (62 %) studies (Table 3). From these 9/13 (69 %) and 4/13 (31 %) reported doses for type I and type II adverse effects respectively. The maintenance doses were within the recommended dose margins in all but one study. Adequate information about the potassium bromide serum levels was reported in 16/21 (76 %) studies (Table 3). From these, 12/16 (75 %) and 6/16 (37 %) reported serum levels for type I and type II adverse effects respectively. The potassium bromide serum levels were higher than the recommended margins in type I adverse effects (7/12, 58 %), while within normal ranges for type II adverse effects (4/6, 67 %).

### Levetiracetam

There was an overall strong level of evidence provided for the levetiracetam safety profile as monotherapy and adjunctive therapy. Six studies [24, 27, 53, 60, 72, 90] presented data about the safety profile of levetiracetam as monotherapy [27, 72] or adjunct to other AEDs (four remaining studies), giving a combined sample size of 129 dogs. One study [53] included both a clinical trial and retrospective case series part.

All the studies reported only type I adverse effects including neurological and gastro-intestinal signs as the most common (Table 4). Specifically, adverse effects most commonly included were vomiting and sedation followed by ataxia and hyperactivity. Less commonly, anorexia, polyphagia, polydipsia followed by polyuria, diarrhoea, aggression, disobedience and attention seeking were reported (Fig. 7). In the monotherapy study, only one episode of vomiting occurred. In one study, no adverse effects were reported.

Adequate data to allow calculations of the prevalence of adverse effects was reported in all studies (Table 4). From these, only two showed >50 % prevalence of adverse effects for the specific period of treatment during which it was conducted. Based on the 95 % CI, the majority of the population experienced adverse effects in none of the studies (Table 4).

Adequate information about the treatment period was reported in all of the studies (Table 4). From these, in 3/7 (43 %) the treatment period was relatively short (<6 months). Adequate information about the doses was provided in all of studies (Table 4). The maintenance doses exceeded the recommended dose range in two studies. Adequate information about the levetiracetam serum levels was reported in 2/7 (29 %) studies (Table 4), but no correlation could be found between serum levels and safety profile. In one study, however [90], there was a correlation between the administered dose and the safety profile; dogs treated with a higher initial dose (i.e. 60 mg/kg) experienced more adverse effects compared to dogs started on lower doses (20 mg/kg) (65 % (95 % CI: 43.6–86.5 %) vs. 34 % (95 % CI: 14.6–53.4 %), p = 0.03).

### Zonisamide

There was an overall weak level of evidence provided for the zonisamide safety profile either as monotherapy or adjunctive therapy. Seven studies [30, 56, 57, 61, 68, 97, 99] presented data about the safety profile of oral zonisamide either as monotherapy (5 studies) [30, 61, 68, 97, 99] or as an adjunct to other AEDs (2 studies) [56, 57], giving a combined sample size of 83 dogs.

Five studies reported type I and type II adverse effects (Table 5). From these, 3/5 (60 %) and 2/5 (40 %) studies reported type I adverse effects for zonisamide monotherapy and adjunctive therapy (mainly to phenobarbital and/or potassium bromide) respectively. Specifically, in all of the studies, adverse effects most commonly included sedation, followed by ataxia, vomiting and decreased serum ALP activity. Less commonly, increased serum ALT and GLDH activity, aggression, anorexia, emaciation and decreased T4 and serum albumin were reported. In adjunctive therapy studies, adverse effects most commonly included sedation, ataxia and increased serum ALP activity, followed by vomiting, increased serum ALT and GLDH activity. In monotherapy studies all the adverse effects occurred at the same frequency; increased serum GLDH and ALT activity were not reported (Fig. 8). Two studies reported type II adverse effects including idiosyncratic hepatotoxicity and mixed acid–base disorder (Table 5).

Adequate data to allow calculations of the prevalence of adverse effects was reported in 4/7 (57 %) studies (Table 5). From these, only one showed >50 % prevalence of adverse effects for the specific period of treatment they conducted. Based on the 95 % CI, the majority of the population experienced at least one adverse effect in 25 % of the studies (Table 5).

Adequate information about the treatment period was reported in all of the studies (Table 5). From these, in 3/6 (50 %) the treatment period was relatively short (<6 months). Adequate information about the doses was provided in all studies (Table 5). The zonisamide maintenance doses exceeded the recommended dose margins in two studies. Adequate information about the zonisamide serum levels was reported in 6/7 (86 %) studies (Table 5), but no correlation could be found between serum levels and the safety profile.

### Primidone

There was an overall weak level of evidence provided for the primidone safety profile either as monotherapy or adjunctive therapy. Sixteen studies [28, 32, 35, 52, 64, 65, 76, 82, 89, 91, 92, 95, 103–105, 113] presented data about the safety profile of primidone as an adjunct to phenobarbital [104] and as a monotherapy agent (remaining studies), giving a combined sample size of 298 dogs.

Twelve studies reported type I adverse effects including gastro-intestinal and neurological signs as the most common (Table 6). Specifically, adverse effects most commonly included increased serum ALP activity, ataxia and polydipsia followed by sedation, hyperactivity, chronic clinical hepatopathy/toxicity, polyphagia, disorientation and then increased serum ALT activity, hind limb weakness and polyuria. Less commonly reported effects included anxiety, tachypnea and increased serum AST, γ-GT, bile acids, SGPT and GLDH activity (Fig. 9). In two studies [32, 76] no adverse effects attributed to primidone were reported. Three studies reported type II adverse effects (Table 6). Specifically, blood dyscrasias (anemia, thrombocytopenia and/or neutropenia) and dermatitis were reported.

Adequate data to allow calculations of the prevalence of adverse effects was reported in 7/16 (44 %) of the studies (Table 6). From these, 3/7 (43 %) showed >50 % prevalence of adverse effects for the specific period of treatment over which it was conducted. Based on the 95 % CI, the majority of the population experienced at least one adverse effect in 3/7 (43 %) studies (Table 6).

Adequate information about the treatment period was reported in 13/16 (81 %) (Table 6). From these, in 6/13 the treatment period was relatively short (<6 months). Adequate information about the dose was provided in 14/16 (88 %) studies (Table 6). From these 11/14 (79 %) and 3/14 (21 %) reported doses for type I and type II adverse effects respectively. The primidone maintenance doses were higher than the recommended dose margins in type I and type II adverse effects in 5/11 (45 %) and 1/3 (33 %) studies, respectively. Adequate information about the primidone (phenobarbital) serum levels was reported in 5/16 (31 %) studies (Table 6). Of these, all reported type I adverse effects. The phenobarbital serum levels were higher than the recommended margins in 3/5 (60 %) studies.

### Gabapentin

There was an overall weak level of evidence provided for the gabapentin safety profile. Two studies [58, 59] presented data about the safety profile of oral gabapentin as an adjunct to other AEDs, giving a combined sample size of 28 dogs.

Both studies reported only type I adverse effects, including neurological signs as the most common (Table 7). Specific adverse effects most commonly included sedation and ataxia. Less commonly reported adverse effects were polyphagia, pancreatitis, chronic clinical hepatopathy/toxicity, polydipsia, polyuria and increased ALP activity.

Both studies showed >50 % prevalence of adverse effects for the specific period of treatment during which they were conducted (Table 7). Based on the 95 % CI, the majority of the population experienced at least one adverse effect in one study (Table 7). In both studies the treatment period was relatively short (<6 months). The gabapentin maintenance doses were high but within the upper recommended dose margins in one study. Gabapentin serum levels were not measured and, thus, no analysis to detect associations between serum levels and adverse effects was performed.

### Pregabalin

There was an overall insufficient level of evidence provided for the pregabalin safety profile. One study [55] presented data about the safety profile of oral pregabalin as an adjunct to phenobarbital and potassium bromide in 11 dogs.

The study reported only type I adverse effects including neurological signs as the main ones (Table 7). Specifically, sedation, ataxia and increased ALP activity and ALT activity were reported.

The study showed 91 % prevalence of adverse effects for the specific treatment period in which it was conducted. Based on the 95 % CI, the majority of the population experienced adverse effects (Table 7). The treatment period was relatively short (<6 months). No correlation was found between pregabalin dose/serum levels and adverse effects.

### Valproate

There was an overall weak level of evidence provided for the valproate safety profile. One study [62] presented data about the safety profile of sodium valproate in different groups either as a monotherapy or as an adjunct to phenobarbital, primidone or a combination of phenobarbital and phenytoin in 57 dogs. The study reported only type I adverse effects including neurological, gastro-intestinal and dermatological ones (Table 7). Specifically, vomiting, sedation, ataxia and alopecia were reported.

The study did not show >50 % prevalence of adverse effects for the specific period of treatment during which it was conducted. Based on the 95 % CI, the majority of the population did not experience adverse effects (Table 7). The treatment period was unclear. No correlation was found between valproate dose and adverse effects. The serum levels were not measured.

### Felbamate

There was an overall weak level of evidence provided for the felbamate safety profile either as monotherapy or adjunctive therapy. Three studies [47, 70, 78] presented data about the safety profile of felbamate as monotherapy [47] or as an adjunct to phenobarbital in particular and potassium bromide (remaining studies), giving a combined sample size of 22 dogs.

All studies reported type I adverse effects including gastro-intestinal and neurological signs as the most common (Table 7). Specifically, adverse effects most commonly included ataxia, tremors, vomiting, hypersalivation and increased ALP activity. Less commonly, clinical hepatopathy/toxicity, increased ALT activity, sedation, limb rigidity, leucopenia, lymphopenia, thrombocytopenia and keratoconjunctivitis sicca were reported (Fig. 10).

Two studies reported type II adverse effects (Table 7). Specifically, blood dyscrasias (anemia, thrombocytopenia and neutropenia) and dermatitis were reported.

Adequate data to allow calculations of the prevalence of adverse effects was reported in 2/3 (67 %) of the studies (Table 7) and none showed >50 % prevalence of adverse effects for the specific period of treatment during which they were conducted. Based on the 95 % CI, the majority of the population did not experience adverse effects (Table 7).

Adequate information about the treatment period was reported in 2/3 (67 %) (Table 7) and it was relatively short in one study (<6 months). Adequate information about the dose and serum levels was provided in 2/3 (67 %) studies (Table 7). In both of these the maintenance doses were higher than the recommended dose margins. No correlation was found between serum levels and adverse effects.

### Topiramate

There was an overall weak level of evidence provided for the topiramate safety profile. One study [54] presented data about the safety profile of topiramate as an adjunct to phenobarbital, potassium bromide and levetiracetam in 10 dogs. The study reported type I adverse effects, including neurological and gastro-intestinal as the most common. Specific adverse effects most commonly reported were ataxia, sedation, weight loss and increased ALP and ALT activity. However, these adverse effects could be attributed in part to the co-administered AEDs such as phenobarbital and/or potassium bromide. The prevalence of adverse effects was approximately 50 %. The treatment period was relatively short (<6 months). The maintenance dose was within recommended dose margins (Table 7). Topiramate serum levels were not measured.

### Phenytoin

There was an overall weak level of evidence provided for the phenytoin safety profile either as monotherapy or adjunctive therapy. Nine studies [32, 35, 52, 87, 89, 91, 92, 108] presented data about the safety profile of phenytoin as monotherapy (7 studies) [32, 34, 35, 52, 87, 91, 108] as an adjunct to primidone in particular and/or potassium bromide (4 studies) [32, 89, 91, 92], giving a combined sample of 66 dogs.

Five studies reported type I adverse effects including gastro-intestinal as the most common (Table 7). Specifically, increased serum ALP, ALT, bile acids and chronic clinical hepatopathy/toxicity were most commonly included. Less commonly, increased serum AST, γ-GT, bilirubin and bile acids activity, decreased albumin, anorexia and emaciation were reported (Fig. 11). In the monotherapy group, no adverse effects occurred in two studies [32, 34].

Three studies reported type II adverse effects (Table 7). Specifically, idiosyncratic hepatotoxicity in particular and blood dyscrasias (anemia, thrombocytopenia and/or neutropenia) occurred. Blood dyscrasias (pancytopenia) were reported by the monotherapy study [87].

Adequate data to allow calculations of the prevalence of adverse effects was reported in 4/9 (44 %) of the studies and all but two did not show >50 % prevalence of adverse effects for the specific period of treatment they were conducted. Based on the 95 % CI, the majority of the population experienced adverse effect in two studies (Table 7).

Adequate information about the treatment period was reported in 8/9 (89 %) (Table 7). From these, in 6/8 (75 %) the treatment period was relatively short (<6 months). Adequate information about the dose and/or serum levels was provided in 7/9 (77 %) (Table 7). No correlation could be found between dose and adverse effects. Phenytoin serum levels were not measured.

#### B) Safety profile comparisons between AEDs or between AEDs-placebo

### Phenobarbital monotherapy vs placebo or untreated control

**Fig. 12:**
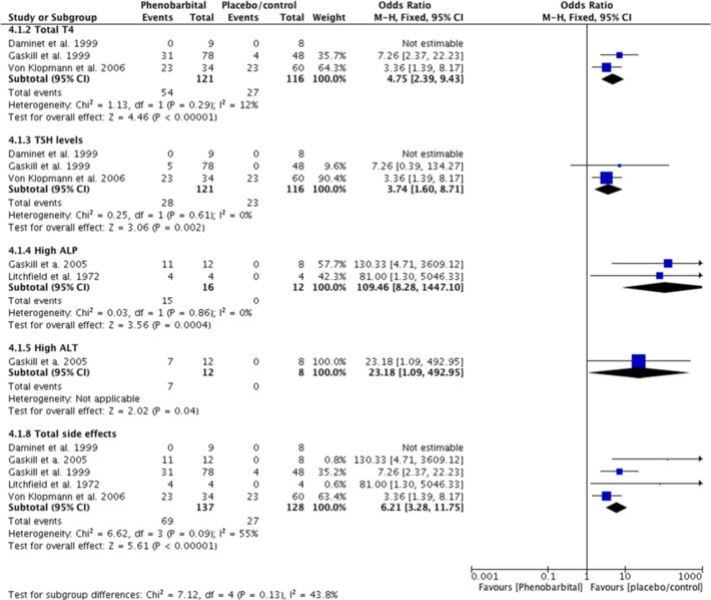
Forest plot comparing phenobarbital vs placebo/control. Odd ratios (95 % CI) of specific and total adverse effects for phenobarbital and control groups

Total safety information was available in five studies [33, 36, 40, 43, 80]. Adequate information to calculate OR was provided in all the studies. Studies compared endocrinal factors and liver enzymes between phenobarbital and untreated or placebo-control groups. Four adverse effects were reported. The common estimated OR was 6.21 (95 % CI: 3.28–11.75), showing a statistically significant association (P < 0.001), with reduced odds of overall adverse effects in the untreated controls. Thus, treated dogs were over 6 times as likely to experience an adverse effect compared to controls. However, moderate heterogeneity was shown between studies (chi^2^ = 6.62, P = 0.09). The OR for abnormal total T4, TSH and increased ALP and ALT activity showed a statistically significant association, with reduced odds of these adverse effects in the controls (Fig. 12).

### Phenobarbital monotherapy vs potassium bromide monotherapy

**Fig. 13:**
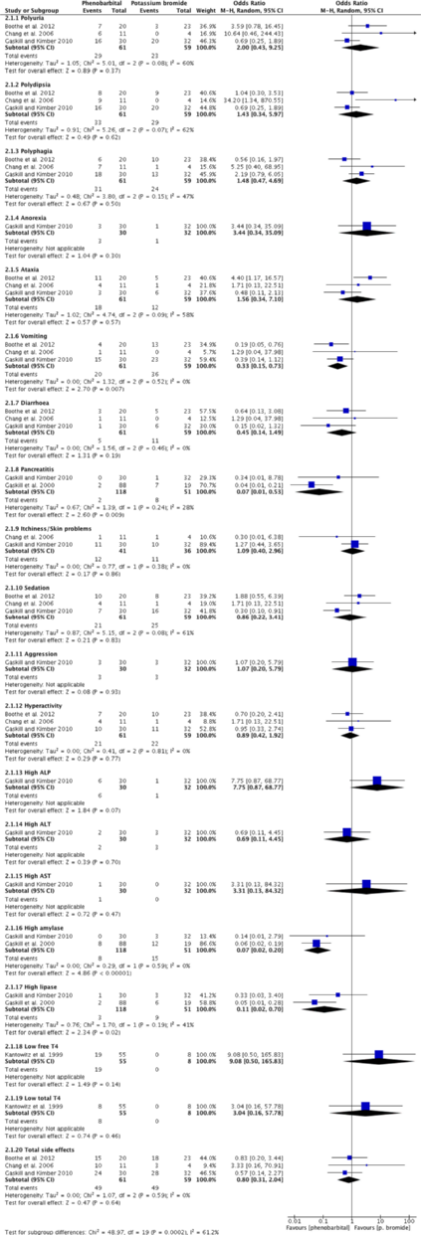
Forest plot comparing phenobarbital vs potassium bromide. Odd ratios (95 % CI) of specific and total adverse effects for phenobarbital and potassium bromide groups

Total safety information was available in five studies [3, 23, 29, 39, 75]. Adequate information to calculate OR was provided in all the studies. Twelve adverse effects were reported. The common estimated OR was 0.80 (95 % CI: 0.31-2.04), showing a statistically non-significant association (P = 0.64) between monotherapies. Low heterogeneity was shown between studies (chi^2^ = 1.07, P = 0.59). The OR for increased serum ALP activity showed a statistical trend in favour of potassium bromide. The OR for pancreatitis, vomiting and increased serum amylase and lipase activity showed a statistically significant association, with reduced odds of these adverse effects in the phenobarbital group (Fig. 13).

### Phenobarbital monotherapy vs levetiracetam monotherapy

**Fig. 14:**
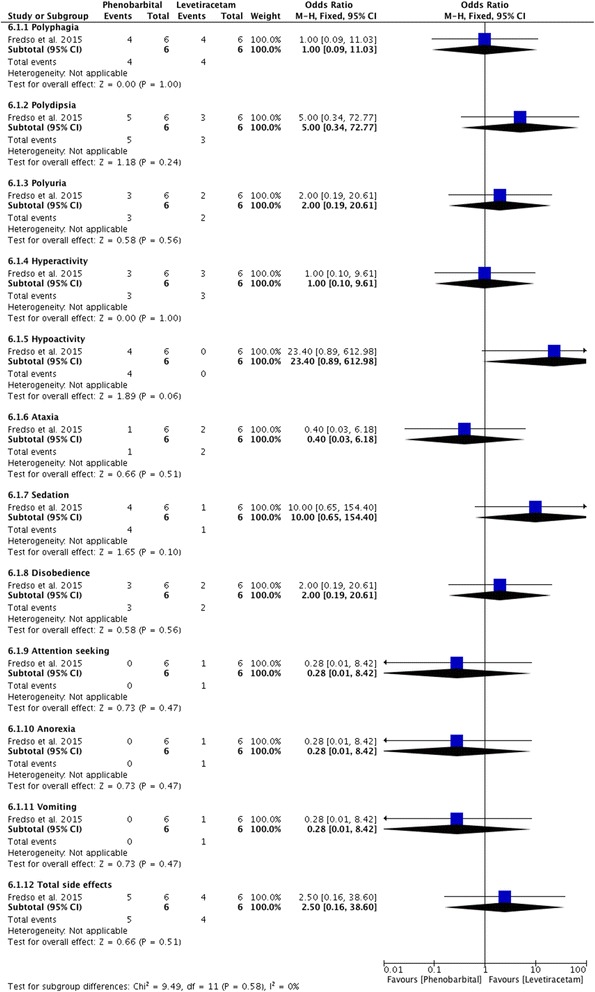
Forest plot comparing phenobarbital vs levetiracetam. Odd ratios (95 % CI) of specific and total adverse effects for phenobarbital and levetiracetam groups

Total safety information was available in one study [27]. Eleven main adverse effects were reported. The common estimated OR was 2.5 (95 % CI: 0.16–38.6), showing a statistically non-significant association (P = 0.51) between monotherapies. The OR for hypoactivity showed a statistically significant association, with reduced odds of this adverse effect in levetiracetam monotherapy (Fig. 14). Although the number of affected dogs did not significantly differ between the groups, the frequency of adverse effects (per dog) was higher in the phenobarbital group in this study.

### Phenobarbital monotherapy vs imepitoin monotherapy

**Fig. 15:**
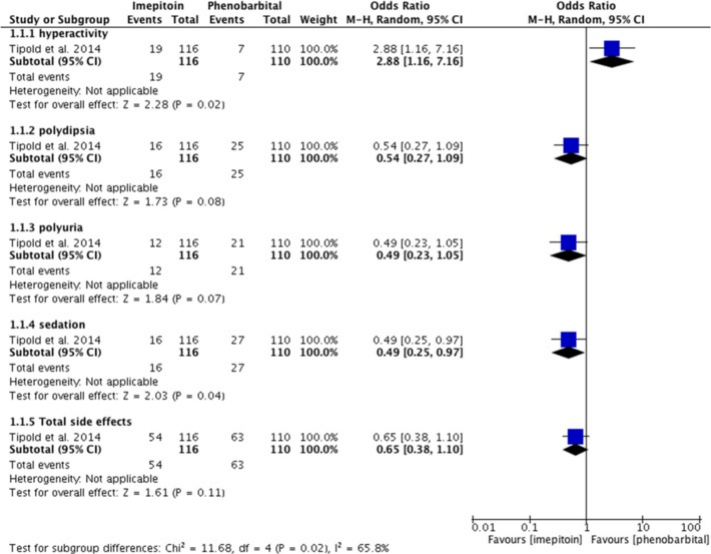
Forest plot comparing phenobarbital vs imepitoin. Odd ratios (95 % CI) of specific and total adverse effects for phenobarbital and imepitoin groups

Total safety information was available in two studies [25, 42]. Adequate information to estimate OR was provided in one study [25] (Fig. 15). Four adverse effects were reported. The common estimated OR was 0.65 (95 % CI: 0.38–1.10), showing a statistically non-significant association (P = 0.11) between monotherapies. The OR for polydipsia and polyuria showed a statistical trend in favor of imepitoin. The OR for sedation and hyperactivity showed a statistically significant association, with reduced odds of these adverse effects in imepitoin and phenobarbital, respectively.

The same study [25] reported that liver enzymes (serum ALP, γ-GT, ALT and GLDH) were increased significantly (P < 0.001) in the phenobarbital group, along with a statistically significant (P < 0.05) association for dose dependence; neither such increase nor dose dependence trend was seen in the imepitoin group.

The second study [42], although the OR could not be estimated, reported sedation, ataxia, polydipsia and polyuria in phenobarbital group, whereas no adverse effects were observed in the imepitoin group.

### Imepitoin monotherapy vs imepitoin adjunctive therapy

**Fig. 16:**
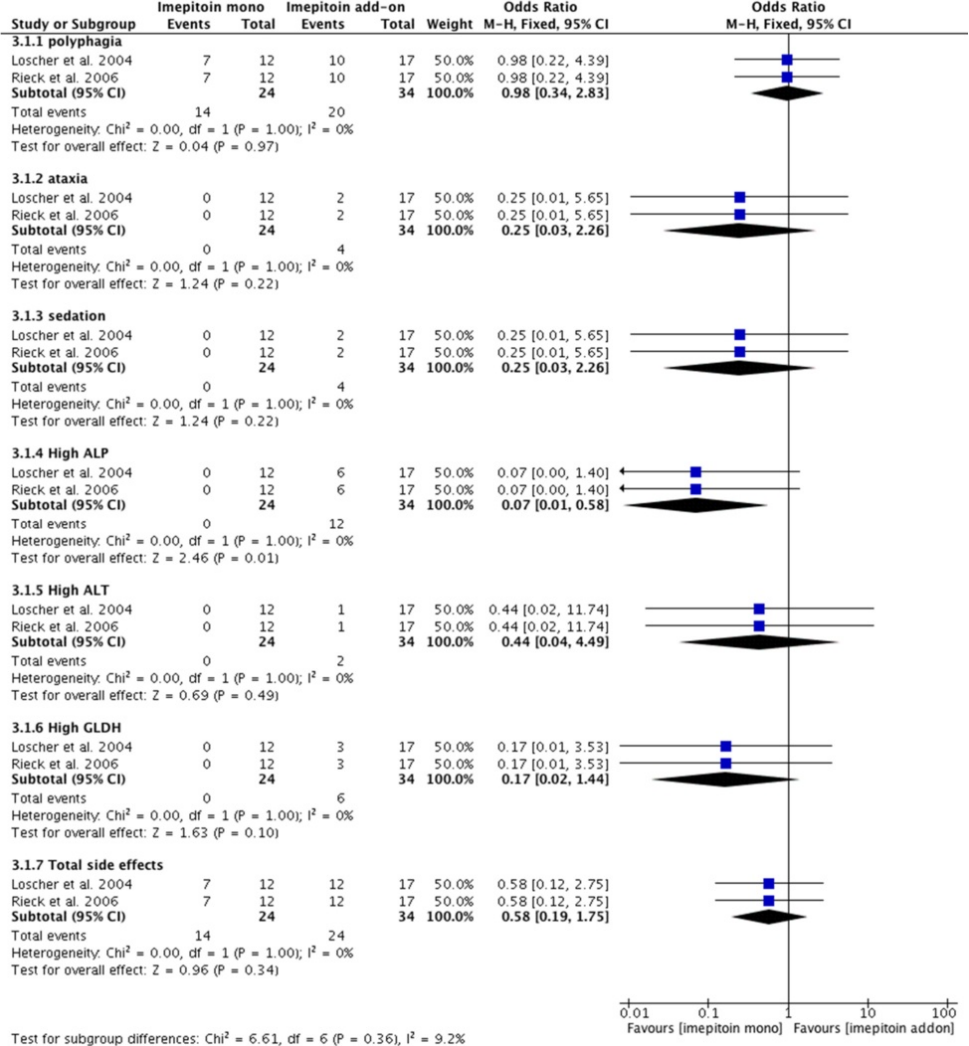
Forest plot comparing imepitoin monotherapy vs imepitoin adjunctive therapy. Odd ratios (95 % CI) of specific and total adverse effects for imepitoin monotherapy and adjunctive therapy groups

Total safety information was available in two studies [44, 48]. Imepitoin monotherapy was compared to imepitoin adjunctive therapy to phenobarbital and/or potassium bromide. Three adverse effects were reported. The common estimated OR was 0.58 (95 % CI: 0.19–1.75), showing a statistically non-significant association (P = 0.34) between the two therapies. No heterogeneity was shown between studies (chi^2^ = 0.00, P = 1.00). The OR for increased serum ALP activity showed a statistically significant association, with reduced odds of this adverse effect in imepitoin monotherapy (Fig. 16).

### Imepitoin monotherapy vs pseudo-placebo

**Fig. 17:**
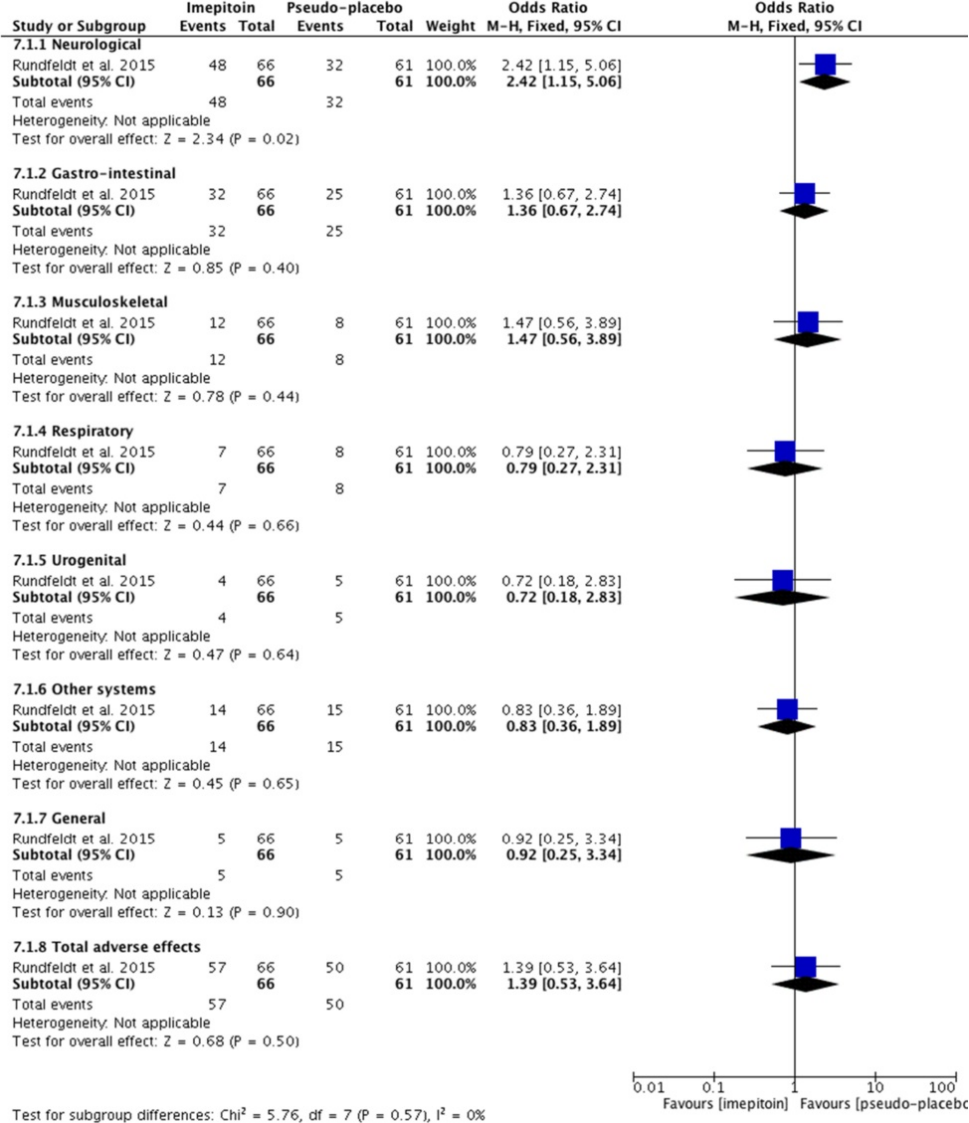
Forest plot comparing imepiton vs pseudo-placebo. Odd ratios (95 % CI) of specific and total adverse effects for imepitoin and control groups

Total safety information was available in one study [26]. Imepitoin monotherapy (high dose) was compared to imepitoin monotherapy (low dose; pseudo-placebo group). The study grouped the adverse effects into broad categories (e.g. neurological, gastro-intestinal, etc.). The adverse effects were reported on the grounds of these categories but they were not specified. The common estimated OR was 1.39 (95 % CI: 0.53–3.64), showing a statistically non-significant association (P = 0.5) between monotherapy and pseudo-placebo. The OR for neurological signs (ataxia and hyperactivity/restlessness) showed a statistically significant association, with reduced odds of this adverse effect in low-dose imepitoin monotherapy (Fig. 17).

The study reported that the findings from the hematology and biochemistry evaluation were unremarkable and remained within normal reference ranges. There was only a very low tendency for serum creatinine activity to increase in the high-dose imepitoin monotherapy compared to low-dose, but this change was also within normal ranges.

### Levetiracetam adjunctive therapy vs placebo

**Fig. 18:**
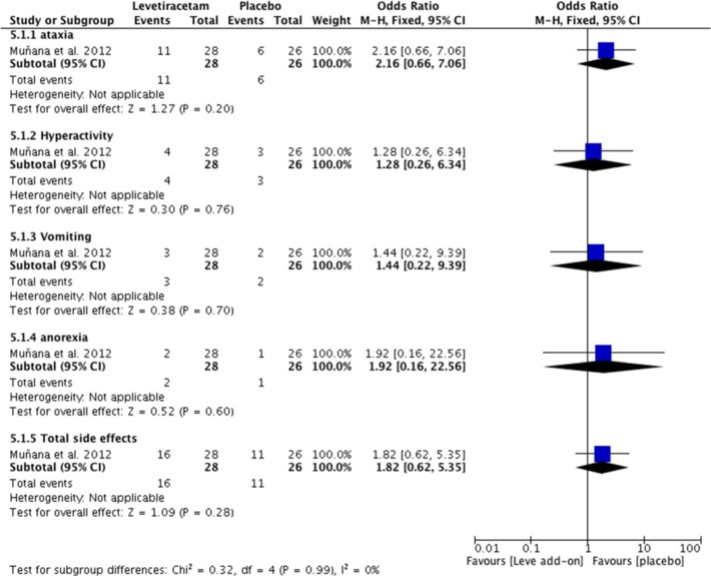
Forest plot comparing levetiracetam vs placebo. Odd ratios (95 % CI) of specific and total adverse effects for levetiracetam and control groups

Total safety information was available in one study [24]. The study compared adverse effects between levetiracetam (as an adjunct to phenobarbital, potassium bromide, gabapentin and/or zonisamide) and placebo-control (as an adjunct to phenobarbital, potassium bromide, gabapentin and/or zonisamide). Four adverse effects were reported. The common estimated OR was 1.82 (95 % CI: 0.62–5.35), showing a statistically non-significant association (P = 0.28) between treatment and placebo (Fig. 18). However, according to the study [24], a significant increase in the prevalence of any of the reported adverse effects (P = 0.013) and ataxia in particular (p = 0.002) was noted in dogs receiving levetiracetam compared to baseline.

Further comparisons between AEDs were not possible due to the lack of comparison studies and/or insufficient report of power analysis and data to allow additional statistical analysis.

## Discussion

To the authors’ knowledge, this is the first systematic review, including a meta-analytic approach, of AEDs’ adverse effects in dogs. The authors followed the PRISMA statement to report this systematic review [120]. The potential for development of AEDs’ adverse effects in dogs are well accepted. Our systematic review found, however, that the strength of evidence as well as the prevalence and occurrence of adverse effects were variable among different AEDs and even among studies evaluating the same AED.

Main categories of adverse effects are type I (pharmacology-related) and type II (idiosyncratic). While type I effects are dose-dependent, occur predictably and are usually caused by a known pharmacological property of the drug, idiosyncratic reactions cannot be explained on the grounds of the drug’s known mechanisms of action and usually occur unpredictably and irrespective of dosage [121]. In our study, type II adverse effects occurred mainly in dogs receiving doses within the recommended margins; however, these adverse effects occurred at any dose. Type II adverse effects occurred in primidone, phenobarbital, potassium bromide and zonisamide. Idiosyncratic reactions were usually caused either by immune-mediated hypersensitivity reactions or by cytotoxic effects of the drug or one of its metabolites [121]. Due to the liver’s central role in drug metabolism it is one of the major sites where idiosyncratic drug reactions manifest. Individual differences in rate of formation and detoxification of reactive metabolites may explain why only certain patients develop idiosyncratic reactions [121]. These unpredictable adverse reactions occur rarely and therefore frequently remain undetected during clinical trials until approval and marketing of drugs. Once a large number of patients is exposed to new or more AEDs, these adverse effects may increase in frequency [122].

This systematic review identified and evaluated ninety studies, the vast majority of which were UCTs, retrospective case series, and reports derived from second and third groups. In total, the data of 4102 dogs were included. Direct and indirect comparisons among drugs based on the frequency, proportion and prevalence of adverse effects in each AED and study showed that levetiracetam and secondary imepitoin might be amongst the safest AEDs, followed by phenobarbital and potassium bromide; a strong level of evidence was identified supporting their safety profile. The remaining AEDs showed a variety of adverse effects, but there was weak evidence to support their safety profile. Direct comparisons could be made only between specific AEDs based on the results of comparison group studies. It was found that imepitoin tended to have a better safety profile than phenobarbital. Levetiracetam appeared to have a better safety profile than phenobarbital, and phenobarbital appeared to have a better safety profile than potassium bromide. However, neither association was statistically significant. The trend for phenobarbital to have a safer profile than potassium bromide supports a former high-quality study which showed that phenobarbital had a better safety profile than potassium bromide [23]. It is interesting that this association was not statistically different in our meta-analysis. It could be that our statistical methods applied were too stringent or that data in our study were more heterogenous, due to the data deriving from various studies, making it less likely in this systematic review to detect a statistical difference. Equally though, it is acknowledged there may be no major underlying biological difference in adverse event rates between these therapies.

Similar to a previous systematic review of AED efficacy [13], the majority of the studies included in this review did not offer high quality of evidence. Studies in the first group, which were considered to offer lower overall risk of bias, were too few compared to other groups (study group 1st:2nd:3rd proportion was 1:4:3). In addition, only 16 and 33 % of studies included well-characterised groups of IE and healthy non-epileptic dogs respectively. Only 13 % of studies included good study population size. Many studies had only a very limited follow-up time to assess efficacy and tolerability (<6 months). Therefore, the results should always be interpreted with caution.

In contrast to AEDs’ efficacy evaluation, case reports do have an important role in forming a safety profile of an individual AED. Specifically, type II or other rare adverse events are unlikely to be recognized by standard clinical trials [123]. Searching the whole range of publications and including NRCTs, UCTs, observational and descriptive studies might avoid missing important information about rare adverse effects and removes potential biases that could occur in systematic reviews which use stricter exclusion criteria and do not include studies other than RCTs [124]. A number of reviews of human drugs have found that case reports and spontaneous reporting systems provide better information about adverse effects than clinical trials do [115, 125]. Indeed, in our review, we found that case reports provided valuable specifics for several kinds of adverse effects not reported in other study designs. Therefore, although small case series and in particular case reports are considered to provide overall low quality evidence for many measurement outcomes (e.g. AEDs’ efficacy), they can reveal valuable information as far as the safety profile is concerned and were included in the current systematic review.

### Difficulties in describing adverse effects among studies

It has been suggested that reporting of AEDs’ adverse events in clinical trials is poor and has not improved over the years [126]. Searching for information about adverse effects can be quite complicated [127–129]. Little evidence exists on the most appropriate searching methods, and a study has assessed different methods in retrieving studies of adverse effects [4]. This study found that a combination of different search methods is required to retrieve as many studies as possible. The same study, however, has also concluded that the most effective search methods might not be possible until there is better reporting and indexing of records concerning the adverse effects. Another recent study has indicated that database searching using adverse effects terms can retrieve the majority of articles (around 92 %) on drugs’ adverse effects [130]. Our review used all the possible searching methods and combinations of these methods and terms in order to retrieve all or the vast majority of the available studies reporting or assessing AEDs’ adverse effects.

The majority of the studies for many AEDs, apart from phenobarbital, primidone, imepitoin and secondary potassium bromide and zonisamide, evaluated the drugs when administered in combination with other AEDs, making it sometimes difficult to determine whether clinical signs could always be attributed to AEDs’ administration. Therefore, should these AEDs be used and investigated more frequently as monotherapy, future analyses (i.e. randomised controlled clinical trials) may allow even clearer conclusions on the safety questions raised in the present systematic review.

In newer standardized trials every sign that occurs during the study will be recorded as an adverse effect even if it is not always associated with the treatment. In addition, newer trials tend to include toxicology studies in which the doses that are administered are sometimes beyond the recommended treatment doses. Therefore, newer trials will report more adverse reactions than others. As imepitoin is a newly developed antiepileptic drug it is possible that the adverse effects have been more adequately captured than for older AEDs and that certain reported adverse effects might have been over-reported or drug unrelated. Furthermore, a few infrequent adverse effects were recorded and mainly occurred in doses higher than the recommended therapeutic ones. The longer a drug is on the market, the likelihood of reports of adverse effects increases. This should be considered when evaluating older AEDs.

The prevalence is a good indicator for evaluating the safety profile of an AED in a study. However, a few limitations originating from the evaluated studies were detected in this review. For instance, in three studies [31, 33, 76], the prevalence was 0 %, but the duration was quite short and only a very small population was included. In another study [75], although a very large population was included, the prevalence was 1 %, possibly because the authors focused on reporting the prevalence of pancreatitis only and the cases were reviewed retrospectively. Similarly, in 23 studies [31, 34–36, 38–40, 43, 45, 46, 49–52, 69, 76, 81, 83–85, 87, 93, 103], the authors focused on reporting only specific adverse effects and, therefore, other adverse effects may have been under- or misreported. In seven studies [36, 81, 83, 85, 87, 89, 104], prevalence was not calculated because only dogs with adverse effects were included. In 16 studies [28, 30, 37, 39, 42, 47, 49, 54, 64, 65, 84, 85, 87, 91, 112, 113], the total number of affected dogs was not very clear and therefore prevalence could not be calculated (risk of prevalence data loses). Lastly, prevalence is difficult to derive from case reports.

There have been some characteristics in the included studies that might have influenced AEDs’ safety profile [1]. First of all, the dose range of a particular AED significantly varied among studies. Another characteristic could be the differences in AEDs’ titration. The safety of some drugs can be influenced by the method and speed of titration [1]. For instance, imepitoin allows quick titration and dose adjustments in dogs with poor response to the drug. However, this was not followed in one study [25] due to the comparison with phenobarbital which demands slow titration. Therefore, this could have negatively affected imepitoin’s safety profile. In addition, the number or frequency of AEDs’ administration could influence safety profiles. Specifically for the AEDs with a short half-life, the number of daily administrations might affect the probability of the occurrence of adverse-effects that correlate to fluctuations of blood levels. However, it has been suggested that this characteristic is not as important as the titration speed [1].

The duration of the study should be sufficient to allow the most frequent adverse effects to occur. In many studies, the relatively small treatment period provided limited time for some adverse effects to occur. Durations of the studies were very heterogeneous and thus the spectrum of the observed adverse effects might have been affected by it. However, one human study [1], which evaluated the AEDs’ central nervous system adverse effects, found that most of them occurred soon after the beginning of treatment or shortly after a dose increase. In our review, adverse effects occurred throughout the treatment duration of the studies.

As was found in the previous systematic review of AEDs’ efficacy [13], several aspects may have also adversely affected the assessment of the reviewed studies. Similarly, the main aspects in the current systematic review were the difference of baseline signalment characteristics, heterogeneity in treatment initiation and protocols between studies, range of study publication dates, publication bias, the several sources of biases related to the studies, the lack of high quality evidence studies (i.e. bRCTs and bRELAS), lack of studies designed with primary aim to investigate AEDs’ safety profile and enrolment of relatively small numbers of animals.

In our study, although the safety profile for each individual AED could be reported and assessed, there were factors that limit definite conclusions on safety profile among AEDs. In a few of the human systematic reviews and/or meta-analysis on AEDs’ safety profile, statistical methods (e.g. comparisons of odds ratio based on study’s evaluated AED and the control group, difference risk ratio, etc.) were conducted for contrasting and combining results from different groups and studies with the aim to identify similar patterns and sources of disagreement among study results or other interesting relationships that may have come to light in the context of multiple studies. In veterinary medicine, though, due to the small number of comparison (i.e. control AED or placebo) clinical trials or ELAS, lack of standardized descriptions of adverse events, variation in methods for data collecting, significant differences among study designs, several potential sources of bias and the fact that objective quantifiable measures and severity of most complaints were not considered in reports, a rigorous statistical method analysis cannot widely be used and, therefore, a meta-analytic approach is very difficult or even impossible. However, in our study, we were able to perform a statistical analysis and meta-analytic exploration for comparison groups within individual studies as well as among a few studies retrieved, which allowed us to evaluate and compare the safety profile between specific AEDs. Only a few more reliable comparisons about the safety profiles could be made, mainly, among phenobarbital, imepitoin and potassium bromide.

### Implications for research

The report and assessment of the safety profile of individual AEDs on systematic reviews is very important, but it is the authors’ opinion that the assessment and comparison of the safety profile among AEDs through a meta-analytic approach could offer even more valuable information and facilitate the clinician’s decision on which AED to choose in respect to its safety profile. Although the prevalence of adverse effects in each study provides a general indicator of each AED’s safety profile and allows limited and indirect comparisons between AEDs, the statistical analysis of comparison group studies is the essential factor that allows direct and more established comparisons. Therefore, further comparison studies are widely needed in order to perform a larger scale meta-analytic study that would offer stronger conclusions and valuable information on which AED can be considered the most or least safe. Towards this goal further conduction of high quality controlled studies (bRCTs and bRELAS) for type I adverse effects and official report of type II adverse effects are vital.

Last, but not least, a further problem that was detected by the evaluation of the studies in this review was the lack of detailed or clear information. This resulted in difficulties when performing statistics for AEDs’ safety profile comparisons. Therefore, it is essential that future studies include as much accurate information as possible and that scientists have wide access to results of clinical trials and experimental studies.

### Implications for clinical practice

It was found that levetiracetam might be one of the safest AEDs based on individual drug assessments, which was also supported by a strong level of evidence. Although phenobarbital is believed to be more commonly associated with a higher adverse effects rate compared to other AEDs such as levetiracetam or imepitoin, it could be argued that it has been longer on the market and a greater number of studies have involved phenobarbital; therefore the number of adverse event reports is likely to be higher. The current evidence based on this systematic review and the previous one [13] shows that there is good level of evidence supporting phenobarbital as one of the most effective AED, but it might have a lower safety profile than other AEDs (i.e. levetiracetam and imepitoin). There was, however, no statistically significant association between the overall safety profile of phenobarbital and imepitoin or levetiracetam. Only for sedation, hypoactivity and hyperactivity, a statistically significant difference was detected in favour of imepitoin, levetiracetam and phenobarbital respectively. Phenobarbital was not compared to further AEDs because of the lack of data (i.e. comparison studies). The remaining AEDs showed a variable safety profile that, potentially, could be high or low; no evidence neither was identified to support any of these statements, nor to compare their safety to other AEDs.

In general, most of the adverse effects reported in all AEDs, apart from the idiosyncratic ones, were not usually life threatening and subsided once doses and serum levels were reduced or following complete AED withdrawal. It is important for clinicians to be able to evaluate the benefits (i.e. the efficacy, cost and frequency of administration, pharmacokinetic properties, need for blood monitoring and lack of possible drug interactions) and risks (i.e. the potential prevalence and severity of adverse effects, long-term impact of adverse effects on patient and owner’s quality of life, drug interactions) before initiating treatment with a specific AED.

## Conclusion

This systematic review provides an evidence-based evaluation of the data on the AEDs’ adverse effects most common usage for canine IE. Case reports were included to ensure that this review would capture idiosyncratic or other rare adverse effects. Only very few studies were designed in a randomised, controlled, and blinded manner and many of the studies included only a small study population with unclear inclusion or exclusion criteria and short term follow-up. Direct comparisons suggested that imepitoin and levetiracetam might have a better safety profile than phenobarbital, whilst the latter might have a better safety profile than potassium bromide. None of the comparisons showed a statistically significant difference. Further comparisons for all the AEDs were not possible as a considerable amount of studies did not report power calculations or clear and adequate data to allow further statistical analysis. Individual AED assessments showed that levetiracetam might be one of the safest AEDs followed by imepitoin and then phenobarbital and potassium bromide; all supported by strong level of evidence. The safety profile in other AEDs was variable but insufficient level of evidence was found to permit firm conclusions.

## Ethics approval and consent to participate

Not applicable.

## Consent for publication

Not applicable.

## Availability of data and materials

The data supporting our findings is contained within the manuscript.

## Abbreviations

AED(s): anti-epileptic drug(s)
bELAS: blinded experimental laboratory animal studies
BID: bis in die (twice daily)
bRCTs: blinded randomised clinical trials
Chloraz: Chlorazepate
CL: confidence level
CSF: cerebrospinal fluid
CT: computer tomography
CTs: clinical trials
Gaba: Gabapentin
IE: idiopathic epilepsy
Leve: Levetiracetam
m: month(s)
MRI: magnetic resonance imaging
NA: Not Available
nbELAS: non-blinded experimental laboratory animal studies
nbRCTs: non-blinded randomised clinical trials
NRCTs: non-randomised clinical trials
NRELAS: non-randomised experimental laboratory studies
OR: odds ratio
PB: phenobarbital
PBr: potassium bromide
PD: polydipsia
PO: per os
PP: polyphagia
PU: polyuria
SF: seizure frequency
SID: semel in die (once daily)
TID: ter in die (three times daily)
UCTs: uncontrolled clinical trials
UELAS: uncontrolled experimental laboratory studies
w: week(s)
